# POC1A promotes malignant phenotypes in non-triple-negative breast cancer cell models with EMT- and Wnt/β-catenin-related alterations

**DOI:** 10.3389/fonc.2026.1856788

**Published:** 2026-06-09

**Authors:** Jingshuang Song, Fanyu Zeng, Yong Du, Ying Huang

**Affiliations:** Department of Breast and Thyroid Surgery, The First Affiliated Hospital of Guilin Medical University, Guilin, China

**Keywords:** breast cancer, cell migration and invasion, cell proliferation, immunohistochemistry, POC1A, Wnt/β-catenin-related alterations

## Abstract

**Background:**

POC1A has been implicated in tumorigenesis in several malignancies; however, its expression pattern, clinical significance, and biological role in breast cancer remain unclear. This study aimed to investigate the expression of POC1A in breast cancer, evaluate its potential clinical relevance, and explore its role in tumor-associated phenotypes.

**Methods:**

Differential expression of POC1A in breast cancer was analyzed using data from The Cancer Genome Atlas (TCGA) and Gene Expression Omnibus (GEO) databases, and its associations with clinicopathological characteristics and patient prognosis were evaluated. Survival analysis was further performed using the Kaplan–Meier Plotter database. Univariate and multivariate Cox regression analyses were performed to assess the prognostic significance of POC1A, and gene set enrichment analysis was conducted to predict signaling pathways potentially associated with POC1A. Immunohistochemistry was subsequently performed to determine POC1A protein expression in breast cancer tissues and analyze its correlations with clinicopathological features and clinical outcomes. In addition, POC1A knockdown and overexpression models were established in MCF-7 and SK-BR-3 cells, representing luminal and HER2-positive non-triple-negative breast cancer cell models, respectively. Cell proliferation, cell-cycle distribution, migration, and invasion were assessed using CCK-8, colony formation, flow cytometry, and Transwell assays. Western blotting was performed to examine the expression of key molecules involved in the Wnt/β-catenin signaling pathway and epithelial-mesenchymal transition (EMT).

**Results:**

POC1A was significantly upregulated in breast cancer tissues in public datasets and showed potential discriminatory ability between breast cancer and normal breast tissues, with an area under the curve (AUC) of 0.879 in the TCGA cohort and 0.901 in the GSE22820 cohort. High POC1A expression was significantly associated with larger tumor size and advanced clinical stage. In the TCGA cohort, multivariate Cox regression analysis identified high POC1A expression as an independent risk factor for poor prognosis in patients with breast cancer based on the included covariates (HR = 1.049, 95% CI: 1.010–1.089, P = 0.014). Kaplan–Meier Plotter analysis further showed that high POC1A mRNA expression was significantly associated with poorer survival outcomes in patients with breast cancer. Immunohistochemical analysis further confirmed elevated POC1A protein expression in breast cancer tissues and its associations with tumor size and clinical stage. However, POC1A protein expression was not validated as an independent prognostic factor in the tissue microarray cohort. Functionally, POC1A knockdown suppressed breast cancer cell proliferation, colony formation, migration, and invasion, whereas POC1A overexpression exerted the opposite effects. At the molecular level, POC1A modulation was associated with changes in Wnt/β-catenin signaling-related proteins and EMT-related markers.

**Conclusions:**

POC1A is overexpressed in breast cancer and may contribute to malignant phenotypes in non-triple-negative breast cancer cell models by promoting cell proliferation, migration, and invasion. These effects may be associated, at least in part, with alterations in Wnt/β-catenin signaling-related proteins and EMT-related markers. POC1A may represent a candidate molecular marker and potential target for further investigation in breast cancer, although its diagnostic, prognostic, and mechanistic significance requires further validation.

## 1 Introduction

Breast cancer is one of the most common malignancies affecting women and is characterized by high incidence, marked heterogeneity, and complex patterns of progression (1). According to GLOBOCAN 2022 data released by the International Agency for Research on Cancer of the World Health Organization, approximately 2.3 million new cases of breast cancer and 670,000 related deaths were reported worldwide among women in 2022, highlighting its substantial burden on public health and clinical care (2). With the widespread implementation of screening programs and advances in imaging and pathological techniques, the detection of early-stage breast cancer has improved markedly, leading to significant therapeutic benefits (3). Nevertheless, a considerable proportion of patients still develop recurrence, metastasis, or treatment resistance during the course of disease management, which severely compromises long-term survival and quality of life (4). Therefore, identifying novel molecular biomarkers associated with breast cancer progression, as well as elucidating their roles and mechanisms in malignant phenotypes such as proliferation, invasion, and metastasis, is of great importance for improving risk stratification, enhancing the precision of therapeutic decision-making, and uncovering potential therapeutic targets.

The proteome of centriole 1A (POC1A) gene encodes a protein closely associated with centrosomal function and is primarily involved in centrosome formation and maintenance (5). The centrosome is a critical cellular organelle that regulates spindle assembly during cell division and ensures accurate chromosome segregation (6). Increasing evidence suggests that centrosomes play a pivotal role in the regulation of cell division and cell-cycle progression (7), and centrosome amplification has been recognized as a hallmark of cancer; its dysfunction may contribute to tumor initiation and progression (8) (9). In recent years, growing attention has been directed toward the role of POC1A in various diseases, particularly malignancies. For example, one study showed that POC1A may promote lung adenocarcinoma progression by regulating the cell cycle and immune cell infiltration, suggesting its potential as a diagnostic and prognostic biomarker and therapeutic target (10). In contrast, Lu et al. reported that POC1A functions as a tumor suppressor in gastric cancer by modulating cell-cycle progression and cellular growth (11). Yuma Wada et al. identified POC1A as an important predictor of recurrence in intrahepatic cholangiocarcinoma (12). Moreover, bioinformatic analyses, including gene expression profiling and mutation analyses, have revealed close associations between POC1A and ovarian, prostate, and colorectal cancers (13) (14) (15). Notably, recent evidence in breast cancer has suggested a potential oncogenic role for POC1A, indicating that it may promote the growth and metastasis of triple-negative breast cancer (TNBC) by activating the signal transducer and activator of transcription 3 (STAT3) signaling pathway and inducing epithelial-mesenchymal transition (EMT) (16). STAT3 signaling has been widely implicated in TNBC progression, metastasis, and therapeutic resistance, further supporting the biological relevance of the POC1A-STAT3 axis in this aggressive breast cancer subtype (17). However, apart from these TNBC-focused findings, the expression patterns, clinicopathological significance, and broader molecular mechanisms of POC1A across different breast cancer subtypes remain insufficiently characterized. In particular, whether POC1A also contributes to malignant phenotypes in non-TNBC contexts, such as luminal and HER2-positive breast cancer models, remains unclear.

Against this background, the present study aimed to systematically evaluate the expression profile and biological functions of POC1A in breast cancer at both the tissue and cellular levels, with particular attention to non-TNBC cellular models and pathway-related molecular alterations. First, bioinformatic analyses based on publicly available databases were performed to compare POC1A expression between breast cancer and normal tissues and to preliminarily assess its associations with clinical characteristics and prognostic indicators. Subsequently, at the tissue level, immunohistochemistry was used to examine POC1A protein expression in breast cancer specimens and to analyze its correlations with clinicopathological parameters, including tumor size, histological grade, clinical stage, and patient prognosis. At the cellular level, MCF-7 and SK-BR-3 cell lines representing luminal and HER2-positive non-TNBC breast cancer cell models, respectively, were used to establish bidirectional POC1A overexpression and knockdown models, enabling a comprehensive evaluation of its effects on malignant phenotypes, including cell proliferation, colony formation, cell-cycle progression, migration, and invasion. Given that the Wnt/β-catenin signaling pathway is an important regulator of tumor progression and has been linked to epithelial-mesenchymal transition in cancer, this pathway was selected for exploratory analysis (18). To explore potential molecular alterations associated with these phenotypes, key components of the Wnt/β-catenin signaling pathway and its downstream targets were examined, together with cell-cycle regulators and EMT-related markers, to evaluate the association between POC1A expression and Wnt/β-catenin signaling-related changes. Collectively, this study sought to clarify the expression characteristics and clinicopathological significance of POC1A in breast cancer and to provide preliminary experimental evidence for its potential molecular mechanisms in promoting tumor progression, thereby offering new insights into risk stratification and the identification of potential therapeutic targets.

## 2 Materials and methods

### 2.1 Data sources, preprocessing, and normalization

The transcriptomic data used in this study were obtained from publicly available databases. RNA-seq data and corresponding clinical information for the TCGA-BRCA cohort were downloaded from the GDC Data Portal (19), including 1,111 breast cancer tissue samples and 113 adjacent normal tissue samples. The external validation dataset, GSE22820, was retrieved from the GEO database (20). This dataset was generated using the Affymetrix Human Genome U133 Plus 2.0 Array (GPL570) platform and included 176 primary breast cancer samples and 12 normal breast tissue samples. Because all data were derived from publicly accessible, anonymized databases, no additional ethical approval was required.

For the TCGA-BRCA cohort, raw gene count matrices were generated using the STAR-Counts workflow. Gene length information was extracted from GENCODE annotation files and then used to convert raw counts into FPKM expression values. After quality control, lowly expressed genes were removed. Batch effects were corrected using the ComBat method, and raw count data were normalized using the DESeq2 package. For the GSE22820 cohort, the pre-normalized expression matrix was downloaded from GEO and further curated. Probe annotation was performed according to the GPL570 platform annotation file, and the average expression value was calculated for genes represented by multiple probes. Genes with low expression levels or a high proportion of missing values were excluded, resulting in the final gene expression matrix used for subsequent analyses. All data processing procedures were conducted using R software (version 4.3.1).

### 2.2 Differential expression analysis of POC1A in breast cancer and normal breast tissues

Following data preprocessing, the expression matrices were organized and converted into numeric format. In the TCGA-BRCA cohort, samples were stratified into tumor and adjacent normal tissue groups according to sample type. After log2(FPKM + 1) transformation, the Wilcoxon rank-sum test was used to assess differences in POC1A expression between the two groups.

For the GSE22820 cohort, the gene expression matrix obtained after preprocessing was similarly categorized into tumor and normal breast tissue groups. The same analytical approach was applied for external validation.

The diagnostic performance of POC1A was evaluated using receiver operating characteristic (ROC) curve analysis. Tissue type was defined as the state variable, and POC1A expression level was used as the test variable. The area under the curve (AUC) and its 95% confidence interval (CI) were calculated using the pROC package, and statistical significance was assessed using the DeLong test. The optimal cutoff value was determined based on the Youden index, and the corresponding sensitivity and specificity were reported. The same analytical pipeline was applied to the external validation cohort. All data analyses and visualizations were performed using R software (version 4.3.1).

### 2.3 Correlation analysis between POC1A expression and clinicopathological characteristics in patients with breast cancer

Patients were stratified according to clinicopathological characteristics, and POC1A expression levels were compared among different groups. For dichotomous variables, the Wilcoxon rank-sum test was used, whereas the Kruskal-Wallis test was applied for variables with more than two categories. A two-sided P value < 0.05 was considered statistically significant.

### 2.4 Survival analysis based on the Kaplan–Meier plotter database

The association between POC1A expression and the prognosis of patients with breast cancer was further evaluated using the Kaplan–Meier Plotter online database (http://kmplot.com/analysis/; accessed on September 20, 2024) (21). The “Breast Cancer” module was selected, and POC1A was entered as the gene of interest. The prognostic endpoints analyzed included overall survival (OS), relapse-free survival (RFS), distant metastasis-free survival (DMFS), and post-progression survival (PPS). The Affymetrix probe 226355_at was used to represent POC1A mRNA expression. Patients were divided into high- and low-expression groups according to the median expression level of POC1A. Survival curves were generated using the Kaplan–Meier method, and differences between groups were assessed using the log-rank test. Hazard ratios (HRs) with 95% confidence intervals (CIs) were obtained from the Kaplan–Meier Plotter database. A P value < 0.05 was considered statistically significant.

### 2.5 Univariate and multivariate cox regression analyses and construction and validation of the prognostic nomogram

Patients were stratified into high- and low-expression groups according to the median POC1A expression level. Univariate Cox proportional hazards regression analysis was performed to evaluate the associations of POC1A expression and clinicopathological variables with patient prognosis. Variables with P < 0.05 in the univariate analysis were subsequently included in the multivariate Cox proportional hazards model to identify independent prognostic factors, and HRs with 95% CIs were calculated.

Based on the independent prognostic factors identified in the multivariate analysis, a prognostic nomogram was constructed using the rms package to predict 1-, 3-, and 5-year survival probabilities. The concordance index (C-index) was used to assess the discriminative ability of the model, whereas calibration curves were generated to evaluate the agreement between predicted and observed outcomes. In addition, ROC curves and the corresponding AUC values were used to further assess the predictive performance of the nomogram.

### 2.6 Gene set enrichment analysis

To explore the potential biological functions and signaling pathways associated with POC1A expression, gene set enrichment analysis (GSEA) based on a pre-ranked gene list was performed. Samples were stratified into high- and low-expression groups according to the median POC1A expression level, and differential expression analysis was conducted between the two groups. A pre-ranked gene list was generated using log2 fold change (log2FC) as the ranking metric. Kyoto Encyclopedia of Genes and Genomes (KEGG) pathway enrichment analysis was performed using the clusterProfiler package, with gene sets obtained from the MSigDB v7.4 C2 curated gene sets collection (c2.cp.kegg.v7.4.symbols).

Gene Ontology (GO) enrichment analysis was performed based on the same ranked gene list to evaluate enrichment across three domains: biological process (BP), cellular component (CC), and molecular function (MF). The gene sets were obtained from the MSigDB v7.4 C5 GO gene sets collection (c5.go.v7.4.symbols).

Enrichment analysis was conducted using permutation testing to calculate the normalized enrichment score (NES), followed by multiple testing correction. A nominal P value < 0.05 and a false discovery rate (FDR) < 0.25 were considered indicative of statistically significant enrichment.

### 2.7 Tissue microarray and clinical data

The tissue specimens used in this study were obtained from two commercial human breast tissue microarray slides. Breast cancer tissues were derived from HBreD140Su07, whereas adjacent normal breast tissues were obtained from HBreD077Su01; both slides were purchased from Shanghai Outdo Biotech Company. Comprehensive clinicopathological data were available for all samples, including age, sex, tissue type, histological grade, tumor stage, survival status, survival time, estrogen receptor status, progesterone receptor status, human epidermal growth factor receptor 2 status, and the Ki-67 index.

After excluding samples with tissue loss or inadequate staining quality, a total of 182 cases were included in the final analysis, comprising 124 breast cancer tissues and 58 adjacent normal tissues. All original sample collection and data use were approved by the ethics committees of the respective source institutions, and informed consent was obtained from all patients. This study was conducted in accordance with the ethical principles of the Declaration of Helsinki.

### 2.8 Immunohistochemical staining

Tissue microarray sections were deparaffinized in xylene and rehydrated through a graded ethanol series, followed by heat-induced epitope retrieval. Antigen retrieval buffer and heating conditions were selected according to the antibody datasheet and the manufacturer’s instructions for the detection kit. Endogenous peroxidase activity was subsequently blocked, and nonspecific binding was minimized by appropriate blocking procedures.

The sections were then incubated overnight at 4 °C with a rabbit polyclonal anti-human POC1A antibody (1:200). The following day, after washing with TBST, the sections were incubated with an HRP-conjugated goat anti-rabbit secondary antibody. Signal visualization was achieved using a 3,3′-diaminobenzidine chromogenic kit. The sections were subsequently counterstained with hematoxylin, dehydrated, cleared, and mounted for microscopic observation and image acquisition. Negative control sections were processed identically, except that TBST was used instead of the primary antibody.

### 2.9 Evaluation of immunohistochemical staining and analysis of clinical correlations

Immunohistochemical staining was semi-quantitatively evaluated using a composite scoring system based on staining intensity × proportion of positive cells. Staining intensity was graded on a scale of 0 to 3, whereas the proportion of positive cells was scored from 0 to 4. The final immunoreactive score was calculated as the product of these two parameters, yielding a total score ranging from 0 to 12. All sections were independently assessed by two observers under blinded conditions, and discrepancies were resolved by joint review. Samples that could not be evaluated because of tissue loss or insufficient assessable material were excluded.

Statistical analyses of the immunohistochemical data were performed using SPSS version 26.0 and GraphPad Prism version 9.0. IHC scores between two independent groups were compared using the Mann–Whitney U test, whereas comparisons among multiple groups were performed using the Kruskal–Wallis test. Associations between categorical variables were analyzed using the chi-square test or Fisher’s exact test, as appropriate. Samples were stratified into high- and low-expression groups according to the median immunoreactive score (IRS) to evaluate the associations of POC1A protein expression with clinicopathological characteristics and patient prognosis. Univariate and multivariate analyses were performed using Cox proportional hazards regression models, and HRs with 95% CIs were calculated. A P value < 0.05 was considered statistically significant.

### 2.10 Cell lines and culture conditions

The human breast cancer cell lines MDA-MB-231, MCF-7, and SK-BR-3, as well as the normal human mammary epithelial cell line MCF-10A, were obtained from the Shanghai Institute of Cell Biology. All cell lines were authenticated by short tandem repeat profiling and confirmed to be free of cross-contamination. Cells were routinely cultured in a humidified incubator at 37 °C with 5% CO_2_. MDA-MB-231 and MCF-7 cells were maintained in Dulbecco’s modified Eagle medium (DMEM) supplemented with 10% fetal bovine serum (FBS) and 1% penicillin-streptomycin. SK-BR-3 cells were cultured in a specialized complete medium provided by Procell, whereas MCF-10A cells were maintained in a Procell-specific complete medium. All subsequent experiments were performed using cells in the logarithmic growth phase. All cell lines were routinely tested for mycoplasma contamination before experiments, and only mycoplasma-free cells were used for subsequent functional assays and molecular analyses.

### 2.11 Design and synthesis of POC1A-targeting siRNAs

Small interfering RNAs (siRNAs) specifically targeting the human POC1A gene, together with a negative control siRNA (si-NC), were designed and synthesized. To enhance gene-silencing efficiency and reduce off-target effects, three distinct siRNAs targeting POC1A were mixed at equimolar concentrations to generate an siRNA pool. The siRNA sequences were as follows:

POC1A siRNA pool (si-POC1A):

siRNA-1: sense, 5′-UCUGGGUACCCAAUGUCAATT-3′; antisense, 5′-UUGACAUGGGUACCCAGATT-3′

siRNA-2: sense, 5′-CAGUGAUGACAAGACUGUUAATT-3′; antisense, 5′-UUAACAGUCUUGUCAUCACUGTT-3′

siRNA-3: sense, 5′-GAUCAUGGAGAGUACAGAAATT-3′; antisense, 5′-UUUCUGUACUCUCCAUGAUCTT-3′

Negative control siRNA (si-NC):

sense, 5′-UUCUCCGAACGUGUCACGUTT-3′; antisense, 5′-ACGUGACACGUUCGGAGAATT-3′

### 2.12 Construction of the POC1A overexpression vector

The coding sequence of the human POC1A gene (NM_015426.5) was selected as the target fragment and cloned into the pcDNA3.1(+) eukaryotic expression vector, generating a 1,224-bp insert. BamHI and EcoRI restriction sites were used for vector construction, and a Kozak consensus sequence (CCGCCACC) was introduced upstream of the start codon (ATG) to enhance translational efficiency.

The recombinant plasmid contains an ampicillin resistance gene for bacterial selection in Escherichia coli and a neomycin resistance gene for selection in eukaryotic cells. After construction, the integrity and accuracy of the inserted sequence were confirmed by bidirectional Sanger sequencing. The purified plasmid had a concentration of 500 ng/µL, with a total yield of approximately 400 µg. An empty pcDNA3.1(+) vector was used as the negative control.

### 2.13 POC1A gene knockdown assay (siRNA transfection)

Gene silencing was achieved using a pooled siRNA targeting POC1A, with a nonspecific siRNA (si-NC) serving as the negative control. MCF-7 and SK-BR-3 cells in the logarithmic growth phase were seeded into culture plates according to the experimental design. When cell confluence reached 60%–70%, transient transfection was performed using Lipofectamine RNAiMAX (Invitrogen, USA). The siRNA and transfection reagent were diluted separately in Opti-MEM medium (Gibco, USA), incubated at room temperature according to the manufacturer’s instructions, and then mixed to form transfection complexes, which were added to the cells. Cells were harvested 48–72 h after transfection. Quantitative real-time polymerase chain reaction (qRT-PCR) was used to assess POC1A mRNA expression in MCF-7 and SK-BR-3 cells. In parallel, Western blotting was performed to evaluate POC1A protein expression and determine knockdown efficiency. A reduction of at least 70% in target protein expression relative to the negative control group was considered indicative of successful gene silencing, after which subsequent functional assays were performed.

### 2.14 POC1A gene overexpression assay (plasmid transfection)

Overexpression experiments were performed using the sequence-verified recombinant pcDNA3.1(+)-POC1A plasmid, with the empty pcDNA3.1(+) vector serving as the negative control. MCF-7 and SK-BR-3 cells in the logarithmic growth phase were selected for plasmid transfection. Cells were seeded into 6-well plates at an appropriate density 24 h before transfection. When cell confluence reached 60%–70%, transient transfection was performed using Lipofectamine 3000 (Invitrogen, USA). A total of 2.5 µg plasmid DNA and 5 µL P3000 reagent were diluted in 125 µL Opti-MEM medium and gently mixed. Separately, 3.75 µL Lipofectamine 3000 was diluted in 125 µL Opti-MEM. The two solutions were then combined and incubated at room temperature for 10–15 min to allow the formation of transfection complexes, which were subsequently added evenly to each well. Cells were harvested 72 h after transfection. qRT-PCR was performed to assess POC1A mRNA expression in MCF-7 and SK-BR-3 cells. In parallel, Western blotting was performed to evaluate POC1A protein expression and determine overexpression efficiency. Cells showing a marked increase in POC1A protein expression compared with the empty vector control group were selected for subsequent functional assays.

### 2.15 qRT-PCR

Total RNA was extracted from cells using TRIzol reagent, and RNA concentration and purity were measured using a NanoDrop spectrophotometer. Qualified RNA samples were subjected to genomic DNA removal and reverse transcription to synthesize cDNA according to the instructions of the PrimeScript FAST RT Reagent Kit with gDNA Eraser. Quantitative real-time PCR was performed using SYBR Green qPCR Master Mix on a CFX96 real-time PCR detection system. ACTB was used as the internal reference gene. Relative gene expression levels were calculated using the 2^−ΔΔCt method. Each sample was analyzed in triplicate, and all experiments were independently repeated at least three times. Primers were synthesized by GenSysBio (China), and detailed primer sequences are provided in **Supplementary Table 1**.

### 2.16 Western blot analysis of POC1A protein expression

1. Protein extraction and sample preparation: Breast cancer cells subjected to different treatments were collected and washed twice with prechilled phosphate-buffered saline (PBS). Cells were lysed in precooled RIPA buffer supplemented with protease and phosphatase inhibitors and incubated on ice for 30 min with intermittent agitation. The lysates were then centrifuged at 12,000 × g for 15 min at 4 °C, and the supernatant was collected as the total protein extract. Protein concentration was determined using a BCA protein assay kit, and sample concentrations were adjusted with RIPA buffer to ensure equal loading across groups. Subsequently, 5×SDS-PAGE loading buffer was added, and samples were denatured at 100 °C for 10 min. Denatured proteins were either used immediately for electrophoresis or aliquoted and stored at −80 °C.
2. Electrophoresis and transfer: Equal amounts of protein (20 µg per lane) were separated on 10% SDS-PAGE gels. After electrophoresis, proteins were transferred onto PVDF membranes (0.45 µm, preactivated with methanol) using a wet transfer system at 100 V for 70 min at 4 °C.
3. Blocking and antibody incubation: After transfer, membranes were blocked with 5% nonfat milk in TBST for 1 h at room temperature. Membranes were then washed three times with TBST (5 min each) and incubated overnight at 4 °C with primary antibodies against POC1A (Zhengneng, 1:1000) and GAPDH. After incubation, membranes were washed three times with TBST (10 min each) to remove unbound primary antibodies, followed by incubation with HRP-conjugated secondary antibodies (ABclonal, 1:10000) for 1 h at room temperature. Membranes were then washed three additional times with TBST (10 min each).
4. Detection and quantitative analysis: Protein bands were visualized using enhanced chemiluminescence reagents and captured using a chemiluminescence imaging system. Target protein expression levels were normalized to GAPDH. Band intensities were quantified using ImageJ software (NIH, USA), and relative expression was calculated as the ratio of target protein to GAPDH (Target/GAPDH). All experiments were independently repeated at least three times. Comparisons between two groups were performed using Student’s t test, whereas comparisons among multiple groups were performed using one-way analysis of variance. A P value < 0.05 was considered statistically significant.

### 2.17 Cell proliferation assay (cell counting Kit-8, CCK-8)

To evaluate the effect of POC1A on the proliferative capacity of breast cancer cells, transient transfection models of POC1A overexpression (OE-POC1A) and knockdown (KD-POC1A) were established. Cell viability was subsequently assessed using the CCK-8 assay (B6005M). MCF-7 and SK-BR-3 cells were divided into the following groups: for the overexpression system, an empty vector control group (MOCK-OE) and a POC1A overexpression group (OE-POC1A); for the knockdown system, a negative control group (MOCK-KD) and a POC1A knockdown group (KD-POC1A).

Cells in the logarithmic growth phase were harvested by trypsinization and resuspended in complete medium containing 10% FBS. Cell density was adjusted to 5×10^4^ cells/mL. Cells were seeded into 96-well plates at 100 µL per well (5×10^3^ cells/well), with six technical replicates per group. Plates were incubated at 37 °C in a humidified atmosphere containing 5% CO_2_. At 0 h (after cell attachment) and 24, 48, and 72 h of culture, 10 µL CCK-8 reagent was added to each well, followed by incubation for 2 h in the dark. The optical density (OD) at 450 nm was then measured using a microplate reader. All experiments were independently performed in triplicate.

### 2.18 Colony formation assay

MCF-7 and SK-BR-3 cells in the logarithmic growth phase were harvested by trypsinization, resuspended in 2 mL complete medium at a density of 2,000 cells per well, and evenly seeded into 6-well plates. Cells were cultured at 37 °C in a humidified atmosphere containing 5% CO_2_. The medium was not changed during the first 5 days; thereafter, 1 mL fresh medium was added every 2–3 days, with a total culture duration of 10–14 days. Once visible colonies had formed, the medium was discarded, and the cells were gently washed twice with PBS and fixed with 4% paraformaldehyde at room temperature for 15 min. Cells were then stained with 0.1% crystal violet at room temperature for 15–20 min, rinsed with PBS to remove excess stain, and air-dried. Images were acquired using a scanner, and colonies with a diameter > 0.5 mm or containing more than 50 cells were counted. The colony formation rate was calculated as follows: colony formation rate (%) = (number of colonies/number of seeded cells) × 100%. Each group included three replicate wells, and all experiments were independently repeated at least three times.

### 2.19 Cell cycle analysis (flow cytometry)

Treated cells were harvested and washed twice with prechilled PBS at 4 °C. Approximately 1 × 10^6^ cells were resuspended in 0.5 mL PBS, and 3 mL precooled 70% ethanol was added dropwise with continuous vortexing to ensure adequate fixation. Cells were fixed at −20 °C for at least 12 h. Before staining, fixed cells were centrifuged and the ethanol was discarded. Cells were washed once with PBS containing 1% bovine serum albumin (BSA) to minimize cell loss and reduce nonspecific background. The cell pellet was then resuspended in propidium iodide (PI) staining solution containing RNase A at a final concentration of 100 µg/mL and incubated at 37 °C in the dark for 30 min. After incubation, samples were filtered through a 300-mesh nylon filter and immediately analyzed by flow cytometry.

For flow cytometric analysis, debris and small cell fragments were first excluded based on forward scatter and side scatter profiles. Single-cell populations were then gated by excluding doublets and aggregates using pulse-area versus pulse-width or pulse-area versus pulse-height parameters. DNA content was analyzed based on PI fluorescence intensity. The proportions of cells in the G0/G1, S, and G2/M phases were calculated using ModFit LT software. The same gating strategy was applied consistently to all experimental groups and biological replicates. All experiments were independently repeated three times.

### 2.20 Cell migration assay

Cell migratory capacity was evaluated using Transwell chambers. MCF-7 and SK-BR-3 cells in the logarithmic growth phase and subjected to the indicated treatments were harvested, resuspended in serum-free medium, and counted. A total of 2×10^4^ cells in 200 µL serum-free medium was seeded into the upper chamber, whereas 650 µL complete medium containing 10% FBS was added to the lower chamber as a chemoattractant. Plates were incubated at 37 °C in a humidified atmosphere containing 5% CO_2_ for 24 h. After incubation, the inserts were removed and washed twice with PBS. Nonmigrated cells on the upper surface of the membrane were gently removed using a cotton swab. Cells that had migrated to the lower surface were fixed and stained with 0.1% crystal violet solution containing methanol for 20 min, followed by washing with PBS. Migrated cells were observed and imaged under a microscope, and the number of migrated cells was quantified using ImageJ software. Each group included three replicate wells, and all experiments were independently repeated three times.

### 2.21 Cell invasion assay

Cell invasive capacity was assessed using a Transwell invasion assay. Transwell chambers with an 8.0-µm pore size were precoated with Matrigel. Matrigel was diluted 1:8 according to the manufacturer’s instructions, evenly applied to the upper chamber membrane, and incubated at 37 °C for 30 min to allow formation of a reconstituted extracellular matrix layer. MCF-7 and SK-BR-3 cells in the logarithmic growth phase and subjected to the indicated treatments were harvested, resuspended in serum-free medium, and counted. A total of 2×10^4^ cells in 200 µL serum-free medium was seeded into the upper chamber, whereas 650 µL complete medium containing 10% FBS was added to the lower chamber as a chemoattractant. Plates were incubated at 37 °C in a humidified atmosphere containing 5% CO_2_ for 24 h. After incubation, the inserts were removed and washed twice with PBS. Noninvading cells on the upper surface of the membrane were gently removed using a cotton swab. Cells that had invaded through the membrane to the lower surface were fixed and stained with 0.1% crystal violet solution containing methanol for 20 min, followed by washing with PBS. Invaded cells were observed and imaged under a microscope, and the number of invading cells was quantified using ImageJ software. Each group included three replicate wells, and all experiments were independently repeated three times.

### 2.22 Western blot analysis of Wnt/β-catenin signaling pathway-related proteins

To examine alterations in Wnt/β-catenin signaling-related proteins and EMT-related markers associated with POC1A modulation, Western blotting was performed to determine the expression levels of Wnt3a, β-catenin, TCF7, Axin2, GSK3β, phosphorylated GSK3β, c-Myc, Cyclin D1, E-cadherin, N-cadherin, and Vimentin. Detailed experimental procedures are described in Section 2.16. Protein expression levels were normalized to GAPDH, whereas phosphorylated GSK3β was normalized to total GSK3β. Band intensities were quantified using ImageJ software. The antibodies used in this study are listed in **Supplementary Table 2**.

### 2.23 Statistical analysis

Data distribution normality was assessed before applying parametric tests. Normally distributed data were analyzed using Student’s t-test or analysis of variance, as appropriate. Non-normally distributed data and semi-quantitative data were analyzed using non-parametric tests, including the Mann–Whitney U test, Wilcoxon rank-sum test, or Kruskal–Wallis test. A two-sided P value < 0.05 was considered statistically significant.

## 3 Results

### 3.1 Upregulated expression of POC1A in breast cancer tissues

**Figure 1:**
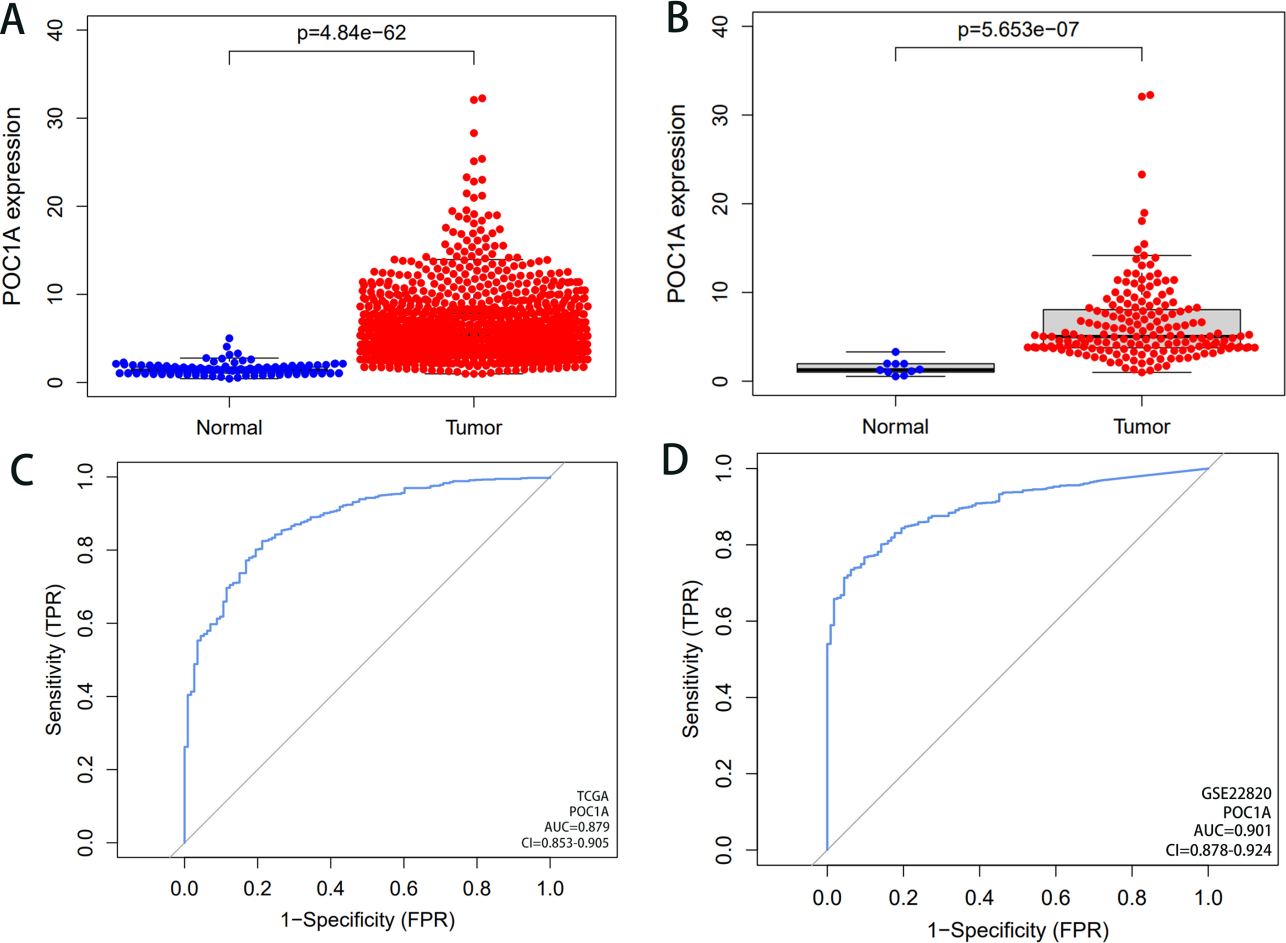
Differential expression of POC1A in breast cancer and normal breast tissues and its discriminatory ability. **(A)** Comparison of POC1A expression levels between breast cancer tissues and adjacent normal breast tissues in the TCGA-BRCA cohort after log2(FPKM + 1) transformation. **(B)** Comparison of POC1A expression levels between breast cancer tissues and normal breast tissues in the GSE22820 cohort. Statistical significance in panels **(A, B)** was determined using the Wilcoxon rank-sum test, and exact P values are shown in the corresponding panels. **(C)** Receiver operating characteristic (ROC) curve analysis of POC1A for distinguishing breast cancer tissues from adjacent normal breast tissues in the TCGA-BRCA cohort. **(D)** ROC curve analysis of POC1A for distinguishing breast cancer tissues from normal breast tissues in the GSE22820 cohort. The area under the curve (AUC) and 95% confidence interval (CI) were calculated using the pROC package, and statistical significance of the AUC was assessed using the DeLong test. ROC, receiver operating characteristic; AUC, area under the curve; CI, confidence interval.

Analysis of the TCGA dataset showed that POC1A expression was significantly elevated in breast cancer tissues compared with normal breast tissues (P < 0.05) (**Figure 1A**). Consistently, the external validation dataset GSE22820 confirmed that POC1A expression was markedly higher in breast cancer tissues than in normal breast tissues (P < 0.05) (**Figure 1B**).

ROC curve analysis suggested that POC1A expression showed potential discriminatory ability between breast cancer tissues and normal breast tissues, with an AUC of 0.879 (95% CI: 0.853–0.905) in the TCGA cohort and 0.901 (95% CI: 0.878–0.924) in the GSE22820 cohort (**Figures 1C, D**).

### 3.2 Correlation between POC1A expression and clinicopathological characteristics in breast cancer

Analysis of TCGA data revealed that POC1A mRNA expression was significantly associated with tumor size (T1 vs. T2, P = 7.3 × 10^-6^; T1 vs. T3, P = 0.004; T1 vs. T4, P = 0.020) and clinical stage (stage I vs. stage II, P = 7.7 × 10^-5^; stage I vs. stage III, P < 0.001). In contrast, no significant associations were observed between POC1A expression and age, sex, lymph node metastasis, or distant metastasis (all P > 0.05) (**Supplementary Figures 1A–F**).

### 3.3 Association between POC1A mRNA expression and prognosis in patients with breast cancer

**Figure 2:**
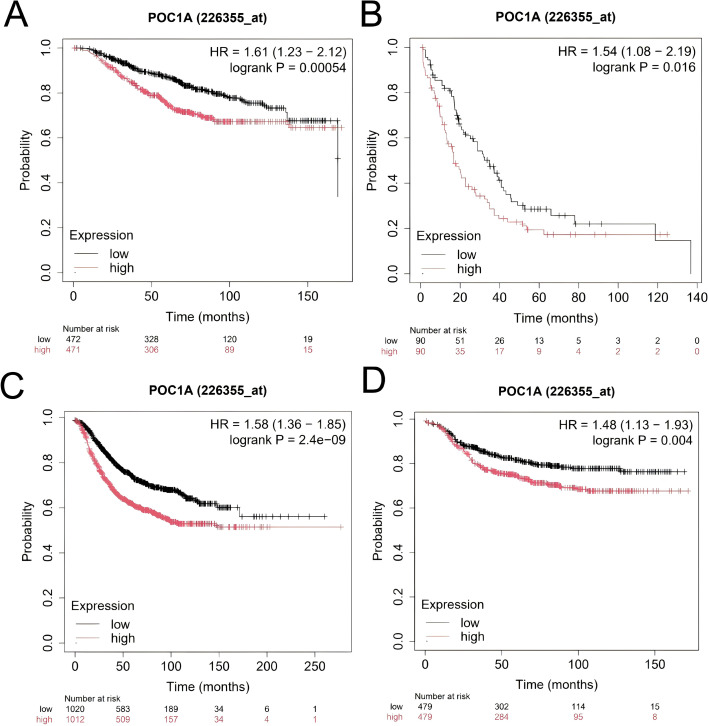
Kaplan–Meier survival analysis according to POC1A mRNA expression in patients with breast cancer. Kaplan–Meier survival analysis was performed using the Kaplan–Meier Plotter online database based on POC1A expression represented by Affymetrix probe 226355_at. Patients were divided into high- and low-expression groups according to the median POC1A expression level. Survival curves are shown for **(A)** overall survival (OS), **(B)** post-progression survival (PPS), **(C)** relapse-free survival (RFS), and **(D)** distant metastasis-free survival (DMFS). The x-axis represents follow-up time in months, and the y-axis represents survival probability. Hazard ratios (HRs), 95% confidence intervals (CIs), and log-rank P values are shown in each panel. Numbers at risk are listed below the survival curves.

Kaplan–Meier Plotter analysis showed that high POC1A mRNA expression, represented by probe 226355_at, was significantly associated with unfavorable prognosis in patients with breast cancer. Specifically, high POC1A expression was associated with worse overall survival (OS; HR = 1.61, 95% CI: 1.23–2.12, log-rank P = 0.001), post-progression survival (PPS; HR = 1.54, 95% CI: 1.08–2.19, log-rank P = 0.016), relapse-free survival (RFS; HR = 1.58, 95% CI: 1.36–1.85, log-rank P = 2.4 × 10^-9^), and distant metastasis-free survival (DMFS; HR = 1.48, 95% CI: 1.13–1.93, log-rank P = 0.004) (**Figures 2A–D**).

### 3.4 Univariate and multivariate cox regression analyses and construction and validation of the prognostic nomogram

#### 3.4.1 Univariate and multivariate cox regression analyses

**Figure 3:**
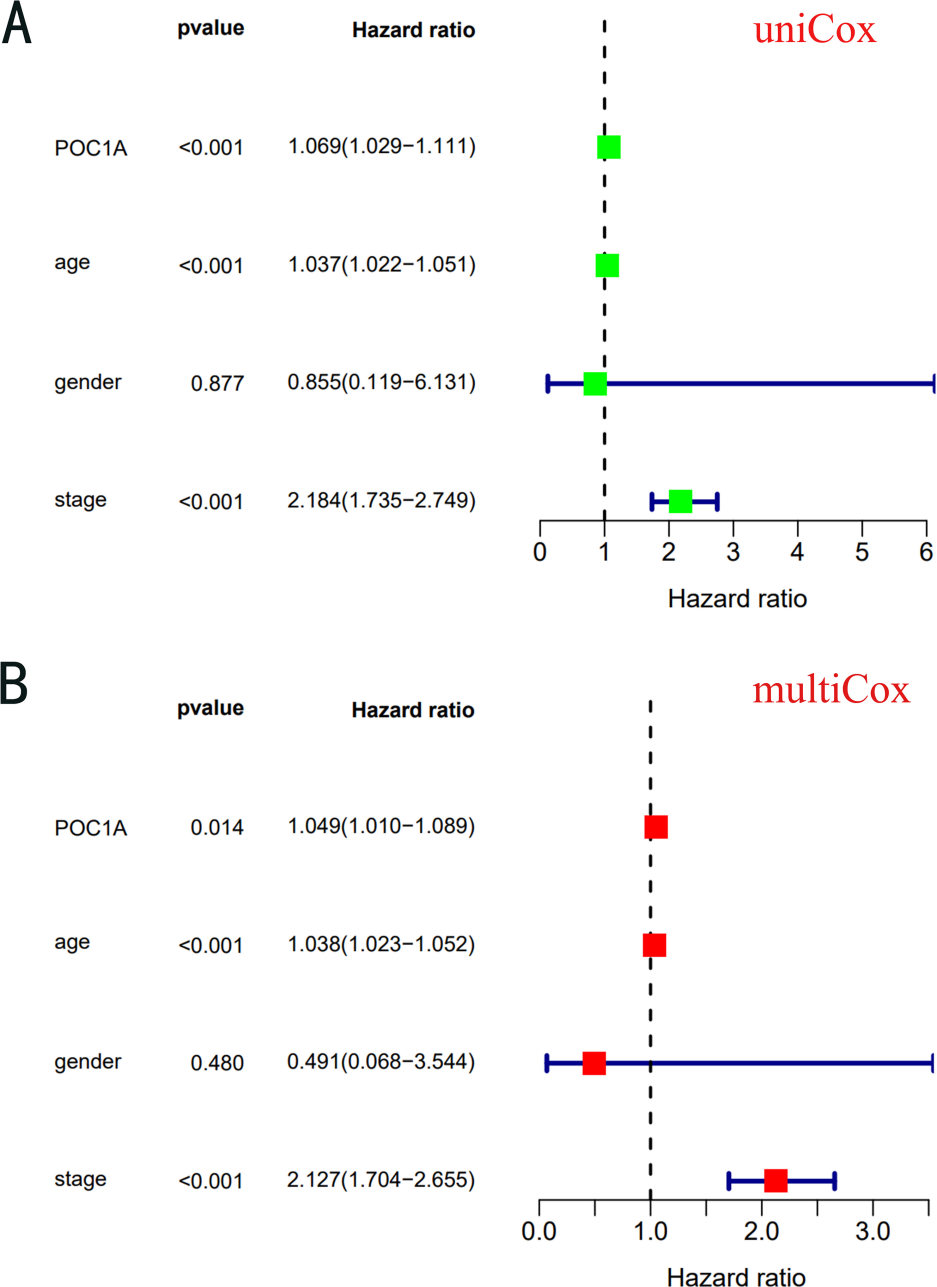
Forest plots of univariate and multivariate Cox regression analyses for overall survival in patients with breast cancer. **(A)** Univariate Cox regression analysis was performed to evaluate the association between each variable and overall survival (OS). **(B)** Multivariate Cox regression analysis was performed to assess the independent prognostic value of POC1A expression after adjustment for age, gender, and clinical stage. Hazard ratios (HRs), 95% confidence intervals (CIs), and P values are shown in the forest plots. The vertical dashed line indicates HR = 1. Variables with P < 0.05 were considered statistically significant.

Univariate Cox regression analysis showed that POC1A expression (HR = 1.069, 95% CI: 1.029–1.111, P < 0.001), age (HR = 1.037, 95% CI: 1.022–1.051, P < 0.001), and tumor stage (HR = 2.184, 95% CI: 1.735–2.749, P < 0.001) were significantly associated with poor prognosis in patients with breast cancer (**Figure 3A**). Multivariate Cox regression analysis further demonstrated that high POC1A expression was an independent adverse prognostic factor in breast cancer (HR = 1.049, 95% CI: 1.010–1.089, P = 0.014) (**Figure 3B**).

#### 3.4.2 Construction and validation of the prognostic nomogram

Based on the multivariate Cox regression model, a prognostic nomogram incorporating sex, age, tumor stage, and POC1A expression was constructed to predict the 1-, 3-, and 5-year survival probabilities of patients with breast cancer (**Supplementary Figure 2A**). Internal validation by resampling yielded a C-index of 0.773 (95% CI: 0.728–0.816), suggesting moderate discriminative performance of the model.

Time-dependent ROC analysis showed AUC values of 0.906, 0.775, and 0.729 for 1-, 3-, and 5-year survival, respectively. Calibration plots showed reasonable agreement between predicted and observed survival probabilities. However, because external validation was not performed, the nomogram should be interpreted as exploratory.

### 3.5 Enrichment analysis reveals POC1A-associated pathways and functions KEGG-GSEA identifies enriched pathways associated with POC1A

**Figure 4:**
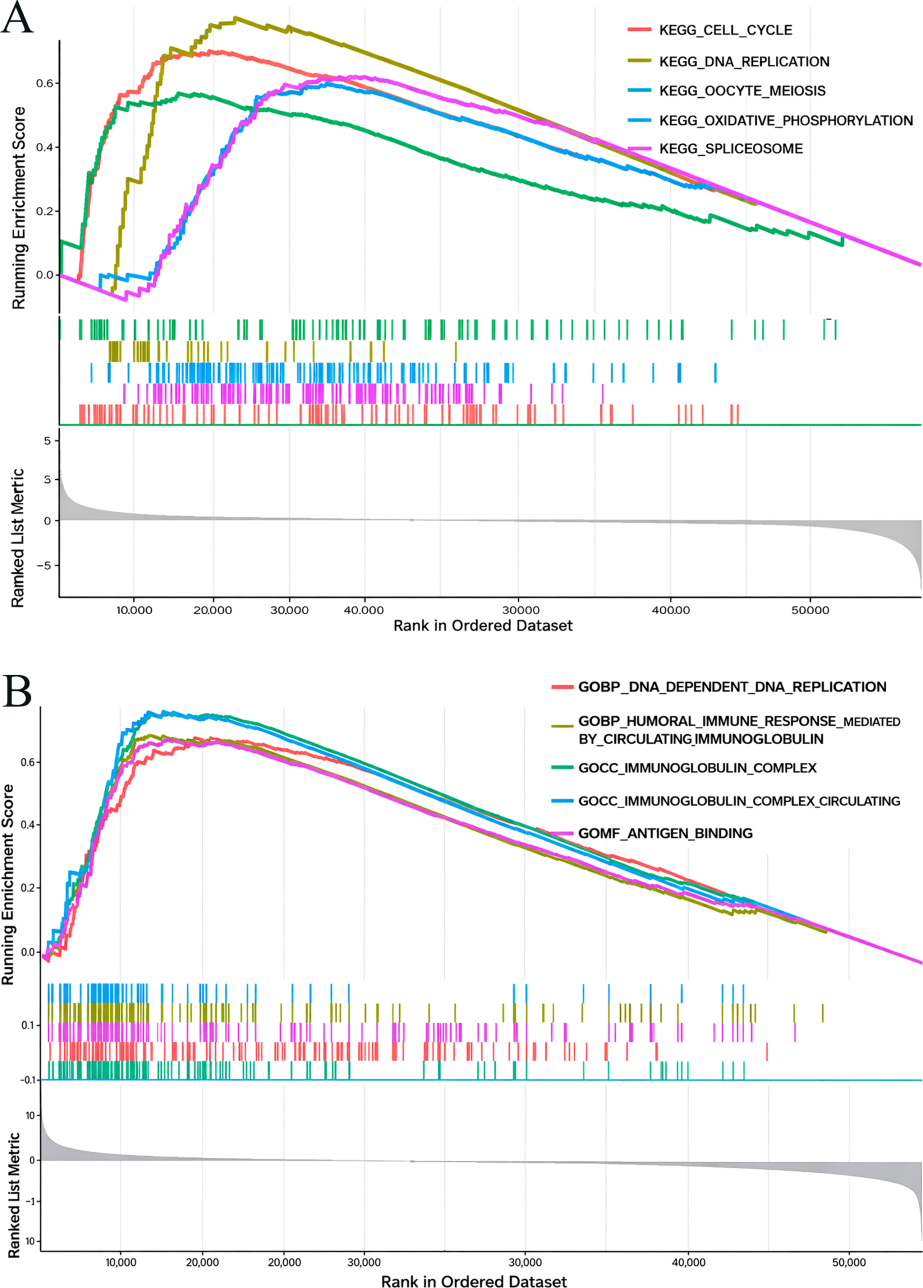
Gene set enrichment analysis of POC1A-associated pathways. **(A)** Kyoto Encyclopedia of Genes and Genomes (KEGG) gene set enrichment analysis (GSEA) comparing the high- and low-POC1A expression groups, showing representative enriched pathways from the MSigDB v7.4 C2 curated KEGG gene set collection. **(B)** Gene Ontology (GO) GSEA comparing the high- and low-POC1A expression groups, showing representative enriched GO terms from the MSigDB v7.4 C5 GO gene set collection. Patients were divided into high- and low-expression groups according to the median POC1A expression level. A pre-ranked gene list was generated using log2 fold change as the ranking metric. Enrichment significance was assessed using permutation testing, and the normalized enrichment score (NES), nominal P value, and false discovery rate (FDR) were calculated. Gene sets with nominal P < 0.05 and FDR < 0.25 were considered significantly enriched.

KEGG-based GSEA demonstrated that the high-POC1A expression group was significantly enriched in multiple pathways involved in tumorigenesis and progression, including the cell cycle, DNA replication, oocyte meiosis, oxidative phosphorylation, and spliceosome pathways (**Figure 4A**).

### 3.6 GO-GSEA indicates enriched biological functions associated with POC1A

GO-based GSEA revealed that the high-POC1A expression group was significantly enriched in biological processes and functional categories related to DNA-dependent DNA replication, circulating immunoglobulin-mediated humoral immune response, immunoglobulin complex, circulating immunoglobulin complex, and antigen binding (**Figure 4B**).

### 3.7 Immunohistochemical validation of elevated POC1A expression in human breast cancer tissues

**Figure 5:**
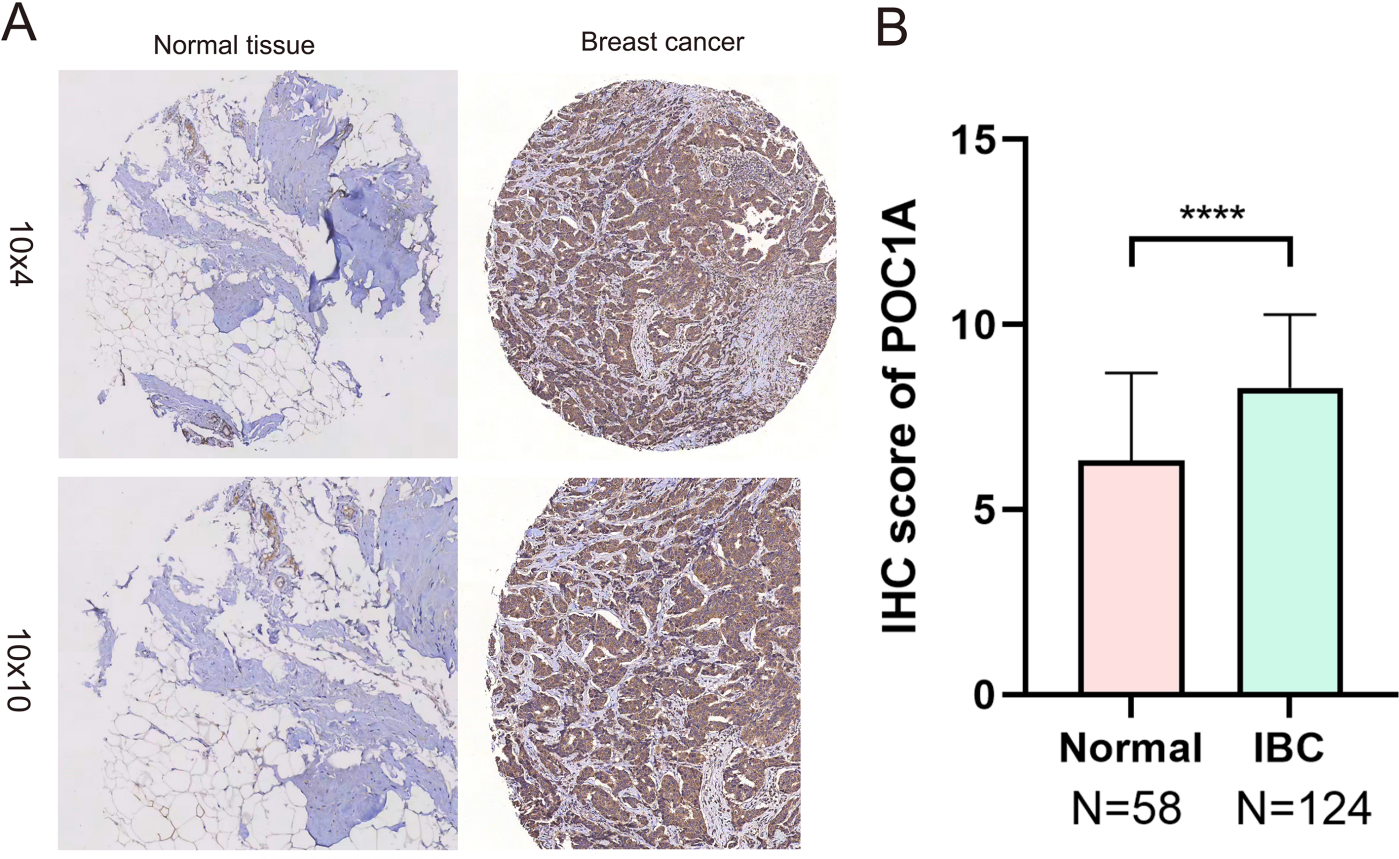
Immunohistochemical analysis of POC1A in breast cancer and normal breast tissues. **(A)** Representative immunohistochemical staining images showing POC1A expression in normal breast tissues and breast cancer tissues at 10×4 and 10×10 magnifications. **(B)** Quantitative analysis of POC1A immunohistochemical scores in invasive breast cancer (IBC) tissues and normal breast tissues. N = 58 for the normal tissue group and N = 124 for the breast cancer group. Data are presented as mean ± SD. Statistical significance was determined using the two-sided Mann–Whitney U test. ****P < 0.0001.

Immunohistochemical analysis showed that POC1A protein expression was significantly higher in breast cancer tissues than in adjacent normal breast tissues (P < 0.05) (**Figure 5**).

### 3.8 Immunohistochemical analysis of the association between POC1A expression and clinicopathological characteristics in breast cancer

Immunohistochemical evaluation revealed that POC1A protein expression was significantly associated with several clinicopathological characteristics in breast cancer. Specifically, POC1A expression differed significantly across tumor size categories, with T1 tumors showing markedly lower expression than T2, T3, and T4 tumors (T1 vs. T2, P = 4.9 × 10^-^¹¹; T1 vs. T3, P = 3.7 × 10^-8^; T1 vs. T4, P = 0.015). Similarly, POC1A expression was significantly lower in stage I tumors than in stage II and stage III tumors (stage I vs. stage II, P = 2.7 × 10^-9^; stage I vs. stage III, P = 5.9 × 10^-9^).

**Figure 6:**
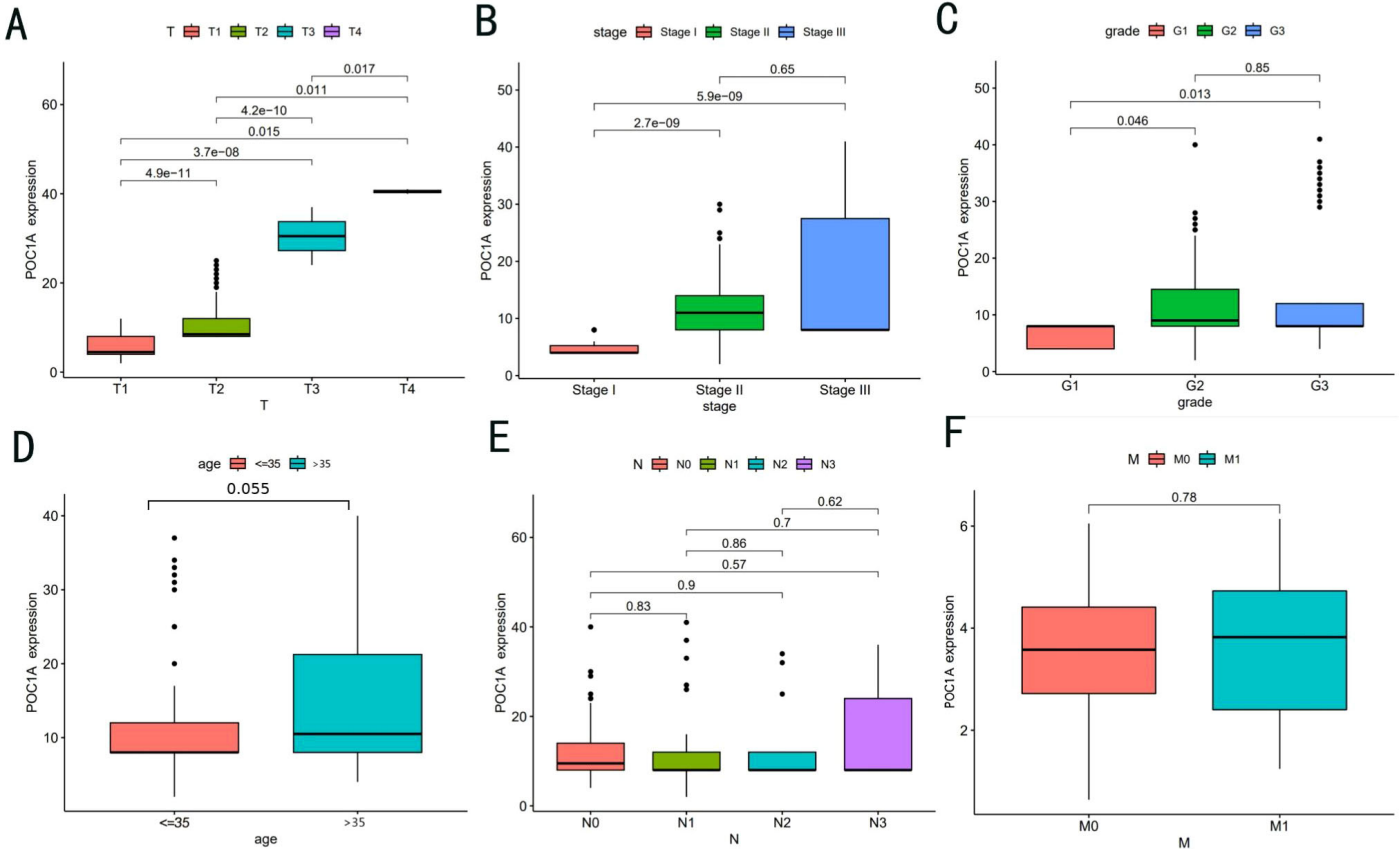
Association between POC1A expression and clinicopathological features in patients with breast cancer. **(A)** POC1A expression across different T stages. **(B)** POC1A expression across different clinical stages. **(C)** POC1A expression across different histological grades. **(D)** POC1A expression between different age groups. **(E)** POC1A expression across different lymph node metastasis groups. **(F)** POC1A expression between different distant metastasis groups. For comparisons among multiple groups in panels **(A–C, E)** statistical significance was assessed using the Kruskal–Wallis test followed by pairwise Wilcoxon rank-sum tests. For two-group comparisons in **(D, F)** statistical significance was assessed using Student’s t-test. Exact P values are shown in the corresponding panels. T, tumor size; N, lymph node metastasis; M, distant metastasis; Stage, clinical stage.

With respect to histological grade, POC1A expression in the G1 group was significantly lower than that in the G2 and G3 groups (G1 vs. G2, P = 0.046; G1 vs. G3, P = 0.013). However, no significant associations were observed between POC1A protein expression and age, lymph node metastasis, or distant metastasis (all P > 0.05) (**Figure 6**).

### 3.9 Immunohistochemical validation of the prognostic value of POC1A in breast cancer

Univariate Cox regression analysis indicated that high POC1A protein expression was significantly associated with poor prognosis in patients with breast cancer (HR = 1.060, 95% CI: 1.030–1.091, P < 0.001). Tumor stage was also significantly associated with adverse prognosis (HR = 2.086, 95% CI: 1.264–3.443, P = 0.004), whereas age (P = 0.680) and histological grade (P = 0.184) were not significantly associated with patient outcomes (**Supplementary Figure 3A**).

Multivariate Cox regression analysis further showed that POC1A protein expression was not an independent prognostic factor in breast cancer (HR = 1.084, 95% CI: 0.943–1.247, P = 0.255), whereas tumor stage remained an independent predictor of prognosis (HR = 1.905, 95% CI: 1.087–3.340, P = 0.024) (**Supplementary Figure 3B**).

### 3.10 Expression of POC1A in breast cancer cell lines and normal mammary epithelial cells

#### 3.10.1 qRT-PCR analysis of POC1A expression in breast cancer cell lines and normal mammary epithelial cells

qRT-PCR analysis showed that POC1A expression was significantly elevated in breast cancer cell lines (MDA-MB-231, MCF-7, and SK-BR-3) compared with the normal mammary epithelial cell line MCF-10A (P < 0.05) (**Supplementary Figure 4A**).

#### 3.10.2 Western blot analysis of POC1A protein expression in breast cancer cell lines and normal mammary epithelial cells

Consistently, Western blot analysis demonstrated that POC1A protein expression was markedly higher in breast cancer cell lines (MDA-MB-231, MCF-7, and SK-BR-3) than in MCF-10A cells (P < 0.05) (**Supplementary Figures 4B, C**).

### 3.11 POC1A knockdown and overexpression experiments

#### 3.11.1 qRT-PCR assessment of knockdown and overexpression efficiency

qRT-PCR was performed to assess POC1A mRNA expression in MCF-7 and SK-BR-3 cells. The results showed a marked increase in POC1A mRNA expression in the overexpression group (OE) and a significant reduction in the knockdown group (KD), whereas no obvious change was observed in the negative control group (NC). These findings confirmed the successful establishment of POC1A overexpression and knockdown models at the transcriptional level (**Supplementary Figure 5**).

#### 3.11.2 Western blot analysis of knockdown and overexpression efficiency

Western blot analysis was performed to assess POC1A protein expression in MCF-7 and SK-BR-3 cells. POC1A protein levels were significantly increased in the OE group compared with the NC group, whereas they were markedly decreased in the KD group. These results further confirmed the successful establishment of POC1A overexpression and knockdown models at the protein level (**Supplementary Figure 6**).

### 3.12 Effects of POC1A overexpression and knockdown on breast cancer cell proliferation (CCK-8 assay)

#### 3.12.1 Effect of POC1A overexpression on cell proliferation

**Figure 7:**
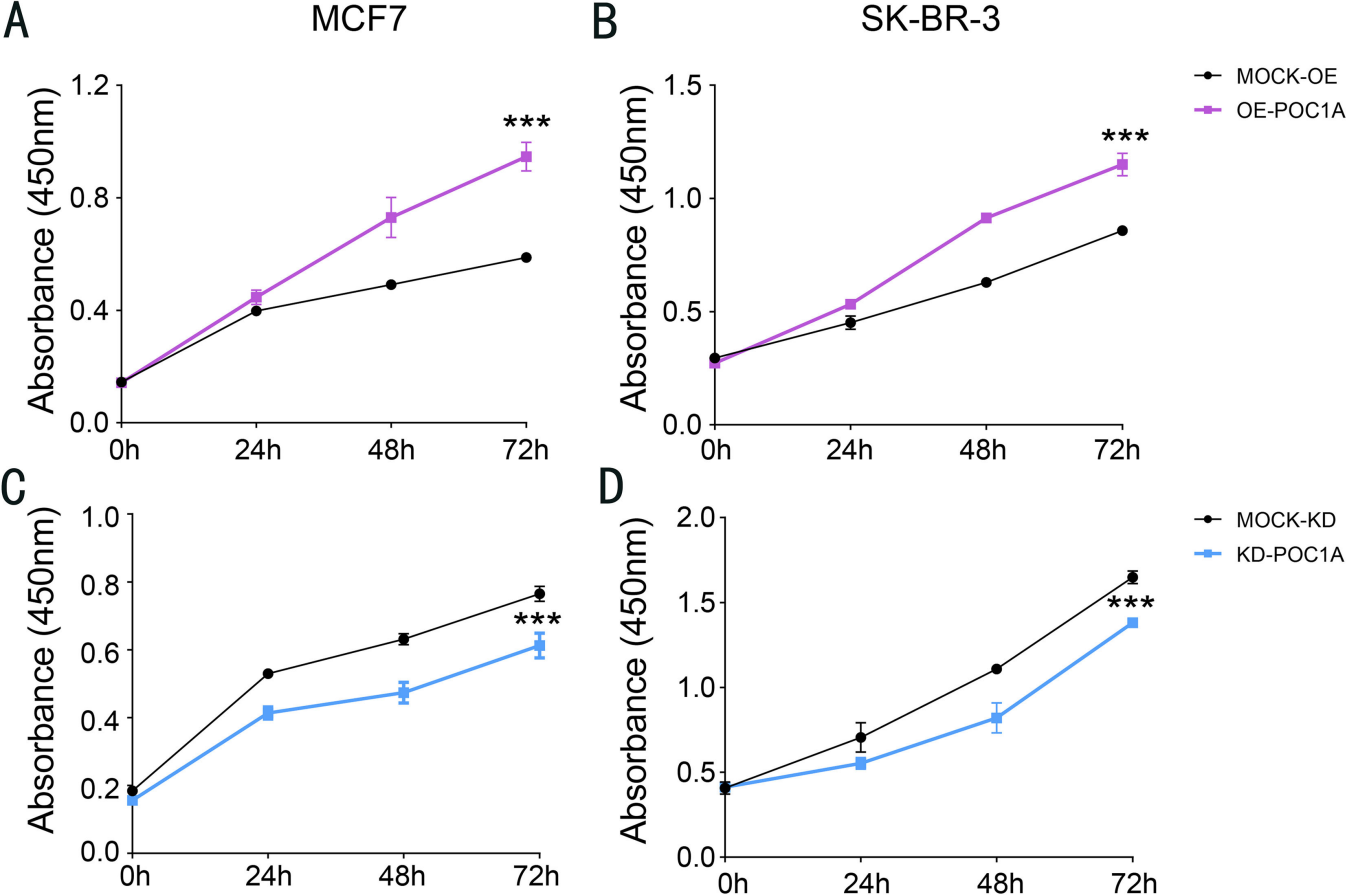
Effects of POC1A overexpression and knockdown on breast cancer cell proliferation assessed by CCK-8 assay. **(A)** Effect of POC1A overexpression on the proliferation of MCF-7 cells. **(B)** Effect of POC1A overexpression on the proliferation of SK-BR-3 cells. **(C)** Effect of POC1A knockdown on the proliferation of MCF-7 cells. **(D)** Effect of POC1A knockdown on the proliferation of SK-BR-3 cells. Cell proliferation was assessed by measuring the optical density at 450 nm at 0, 24, 48, and 72 h. Data are presented as mean ± SD from three independent experiments, with six technical replicates per group in each experiment. Statistical significance was assessed using two-way ANOVA followed by Sidak’s multiple-comparison test. The significance annotations indicate comparisons between the corresponding experimental and control groups at 72 h. ***P < 0.001.

Cell proliferation following POC1A overexpression was assessed using the CCK-8 assay. Compared with the MOCK-OE group, cells in the OE-POC1A group exhibited a markedly increased growth rate. At 72 h, the OD values at 450 nm were significantly higher in the OE-POC1A group (P < 0.001), indicating that POC1A overexpression substantially enhanced the proliferative capacity of MCF-7 and SK-BR-3 cells *in vitro* (**Figures 7A, B**).

#### 3.12.2 Effect of POC1A knockdown on cell proliferation

Cell proliferation following POC1A knockdown was also assessed using the CCK-8 assay. Compared with the MOCK-KD group, cells in the KD-POC1A group showed a significantly reduced growth rate. At 72 h, the OD values at 450 nm were markedly lower in the KD-POC1A group (P < 0.001), indicating that POC1A knockdown significantly suppressed the proliferative capacity of MCF-7 and SK-BR-3 cells *in vitro* (**Figures 7C, D**).

### 3.13 Effects of POC1A knockdown and overexpression on colony formation capacity of breast cancer cells

#### 3.13.1 Effect of POC1A knockdown on colony formation

**Figure 8:**
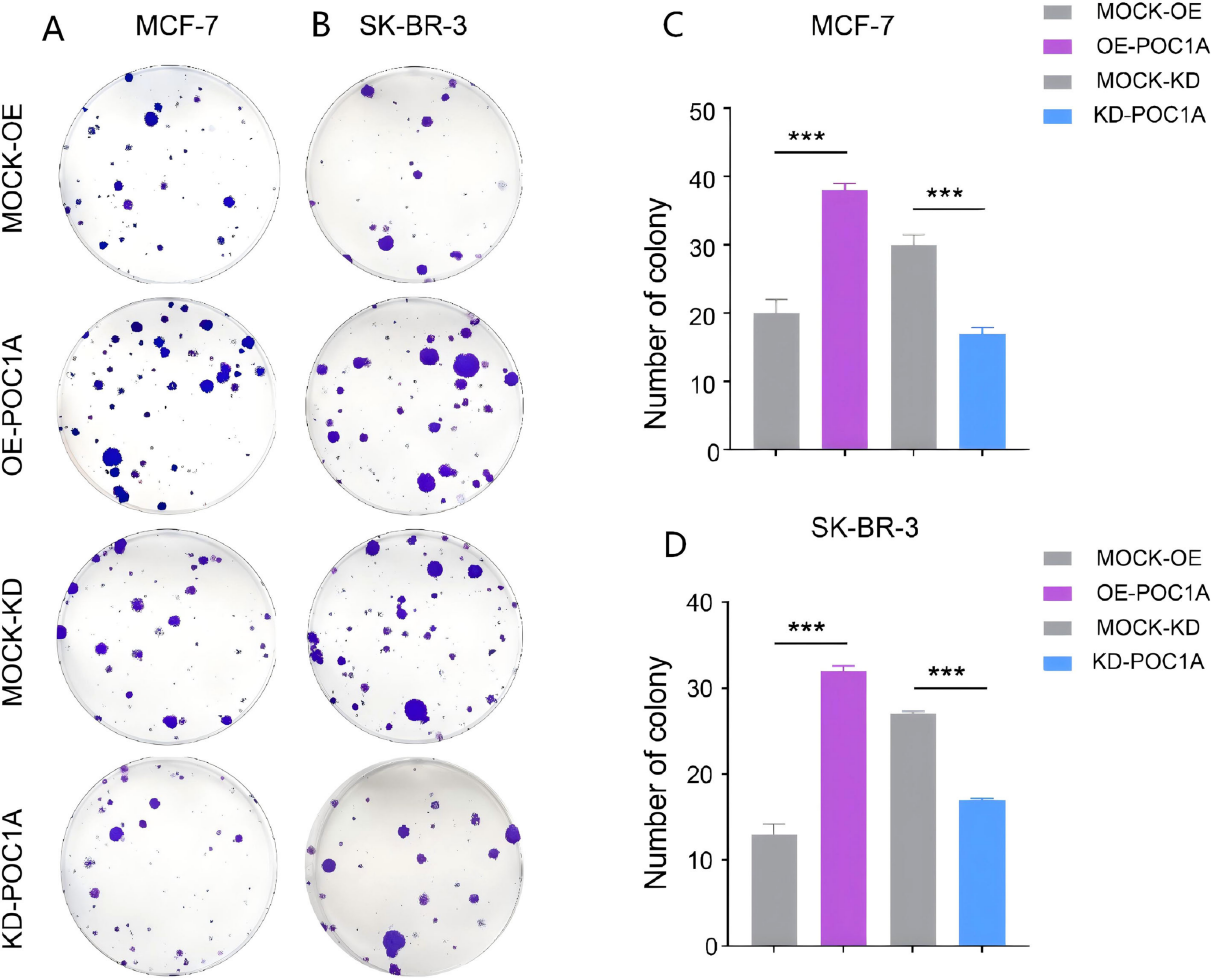
Effects of POC1A overexpression and knockdown on colony formation ability of breast cancer cells. **(A)** Representative images of colony formation in MCF-7 cells with POC1A overexpression or knockdown. **(B)** Representative images of colony formation in SK-BR-3 cells with POC1A overexpression or knockdown. MOCK-OE and MOCK-KD served as the corresponding negative controls for the overexpression and knockdown systems, respectively. **(C)** Quantitative analysis of colony numbers in MCF-7 cells. **(D)** Quantitative analysis of colony numbers in SK-BR-3 cells. Colony formation ability was quantified by counting the number of colonies. Data are presented as mean ± SD from three independent experiments. Statistical significance was determined using an unpaired two-tailed Student’s t-test for each indicated comparison. ***P < 0.001.

Colony formation assays showed that, following POC1A knockdown, the number and size of colonies in the KD-POC1A group were markedly reduced compared with those in the MOCK-KD group. Quantitative analysis further demonstrated that POC1A knockdown significantly decreased the colony formation rate in both MCF-7 and SK-BR-3 cells (P < 0.001). These findings indicate that silencing POC1A impairs the clonogenic capacity of breast cancer cells (**Figure 8**).

#### 3.13.2 Effect of POC1A overexpression on colony formation

Conversely, POC1A overexpression significantly increased both the number and size of colonies in the OE-POC1A group compared with the MOCK-OE group. Quantitative analysis confirmed that POC1A overexpression significantly enhanced the colony formation rate in MCF-7 and SK-BR-3 cells (P < 0.001). These results suggest that POC1A overexpression markedly promotes the clonogenic potential of breast cancer cells (**Figure 8**).

### 3.14 Effects of POC1A knockdown and overexpression on cell cycle distribution in breast cancer cells (flow cytometry)

#### 3.14.1 Effect of POC1A knockdown on cell cycle distribution

**Figure 9:**
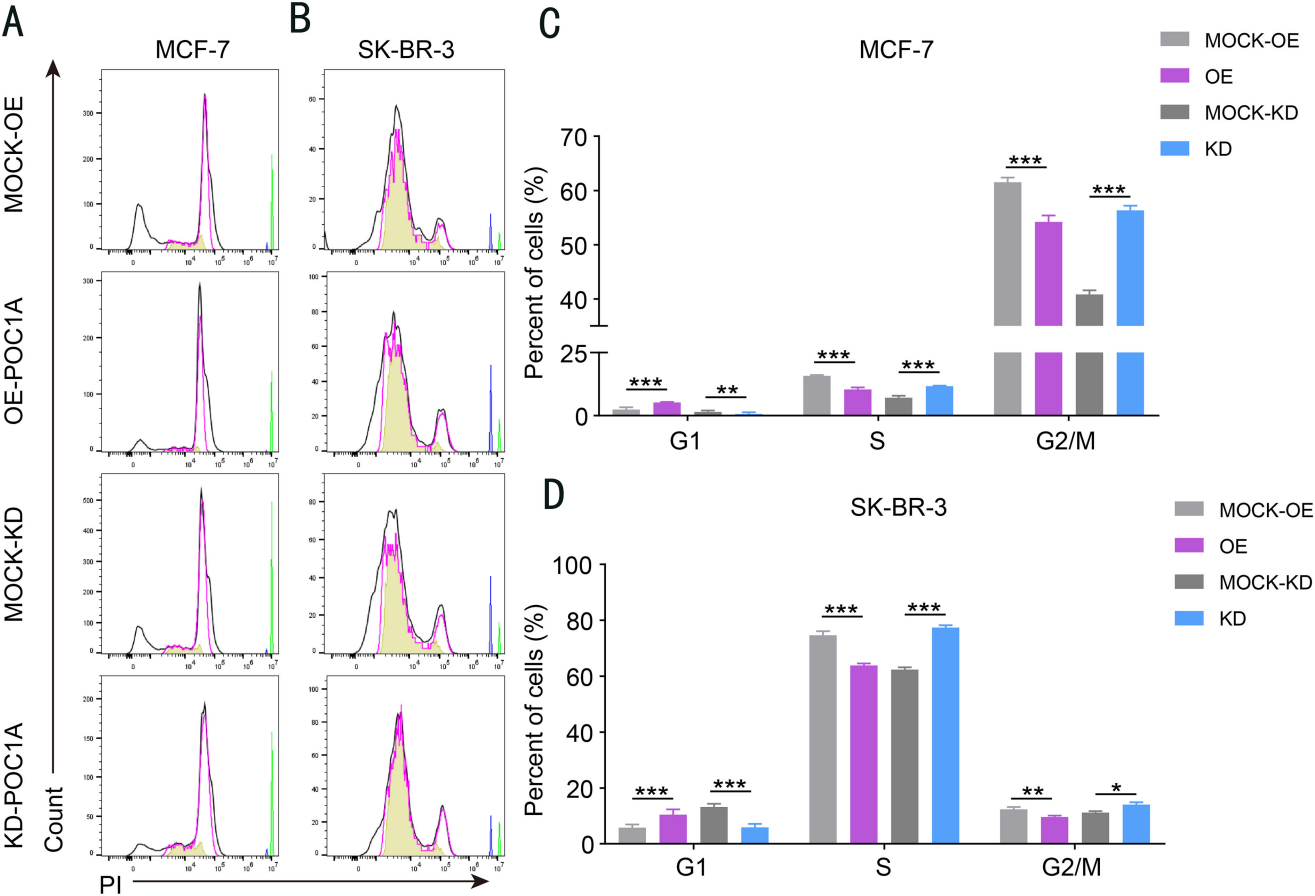
Effects of POC1A overexpression and knockdown on cell-cycle distribution in breast cancer cells. **(A)** Representative flow cytometry histograms showing cell-cycle distribution in MCF-7 cells with POC1A overexpression or knockdown. **(B)** Representative flow cytometry histograms showing cell-cycle distribution in SK-BR-3 cells with POC1A overexpression or knockdown. MOCK-OE and MOCK-KD served as the corresponding negative controls for the overexpression and knockdown systems, respectively. **(C)** Quantitative analysis of the percentages of MCF-7 cells in the G0/G1, S, and G2/M phases. **(D)** Quantitative analysis of the percentages of SK-BR-3 cells in the G0/G1, S, and G2/M phases. Cell-cycle distribution was analyzed by propidium iodide (PI) staining and flow cytometry. Data are presented as mean ± SD from three independent experiments. Statistical significance was determined using an unpaired two-tailed Student’s t-test for each indicated comparison. *P < 0.05, **P < 0.01, ***P < 0.001.

Flow cytometric analysis was performed to evaluate the effect of POC1A knockdown on cell cycle distribution in MCF-7 and SK-BR-3 cells. In MCF-7 cells, compared with the MOCK-KD group, the KD-POC1A group exhibited a marked decrease in the proportion of cells in the G0/G1 phase, accompanied by significant increases in the S and G2/M phases (P < 0.001). Similarly, in SK-BR-3 cells, POC1A knockdown decreased the proportion of cells in the G0/G1 phase and increased the proportions of cells in the S and G2/M phases (P < 0.001) (**Figure 9**).

#### 3.14.2 Effect of POC1A overexpression on cell cycle distribution

Flow cytometric analysis was also performed to assess the effect of POC1A overexpression on cell cycle distribution in MCF-7 and SK-BR-3 cells. In MCF-7 cells, compared with the MOCK-OE group, the OE-POC1A group showed a significant increase in the proportion of cells in the G0/G1 phase, along with marked decreases in the S and G2/M phases (P < 0.001). A similar pattern was observed in SK-BR-3 cells, in which POC1A overexpression increased the G0/G1-phase fraction and decreased the S- and G2/M-phase fractions (P < 0.001) (**Figure 9**).

### 3.15 Effects of POC1A knockdown and overexpression on cell migration in breast cancer cells

#### 3.15.1 Effect of POC1A knockdown on cell migration

**Figure 10:**
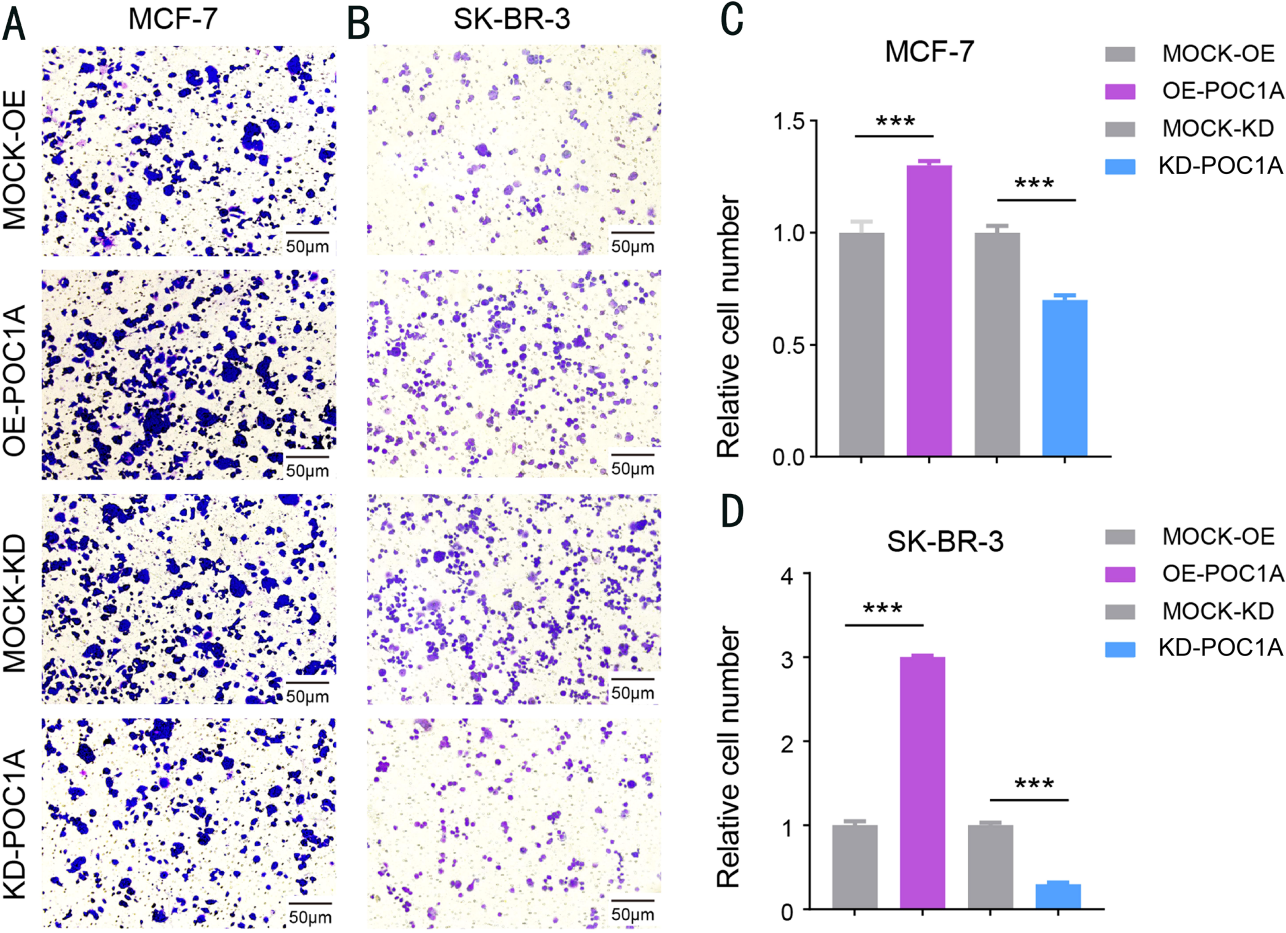
Effects of POC1A overexpression and knockdown on the migration ability of breast cancer cells assessed by Transwell assay. **(A)** Representative images of Transwell migration assays in MCF-7 cells with POC1A overexpression or knockdown. **(B)** Representative images of Transwell migration assays in SK-BR-3 cells with POC1A overexpression or knockdown. MOCK-OE and MOCK-KD served as the corresponding negative controls for the overexpression and knockdown systems, respectively. Scale bar, 50 μm. **(C)** Quantitative analysis of migrated MCF-7 cells. **(D)** Quantitative analysis of migrated SK-BR-3 cells. Migration ability was quantified by counting migrated cells in randomly selected microscopic fields, and the values were normalized to the corresponding MOCK control group. Data are presented as mean ± SD from three independent experiments. Statistical significance was determined using an unpaired two-tailed Student’s t-test for each indicated comparison. ***P < 0.001.

The effect of POC1A knockdown on cell migration was evaluated using a Transwell migration assay. Compared with the MOCK-KD group, the number of MCF-7 and SK-BR-3 cells migrating to the lower surface of the membrane was reduced in the KD-POC1A group. Quantitative analysis demonstrated that the number of migrated cells was significantly decreased in the KD-POC1A group in both cell lines (P < 0.001) (**Figure 10**).

#### 3.15.2 Effect of POC1A overexpression on cell migration

The effect of POC1A overexpression on cell migration was also assessed using a Transwell migration assay. Compared with the MOCK-OE group, the OE-POC1A group exhibited increased numbers of MCF-7 and SK-BR-3 cells migrating to the lower surface of the membrane. Quantitative analysis showed that the number of migrated cells was significantly higher in the OE-POC1A group than in the control group in both cell lines (P < 0.001) (**Figure 10**).

### 3.16 Effects of POC1A knockdown and overexpression on cell invasion in breast cancer cells

#### 3.16.1 Effect of POC1A knockdown on cell invasion

**Figure 11:**
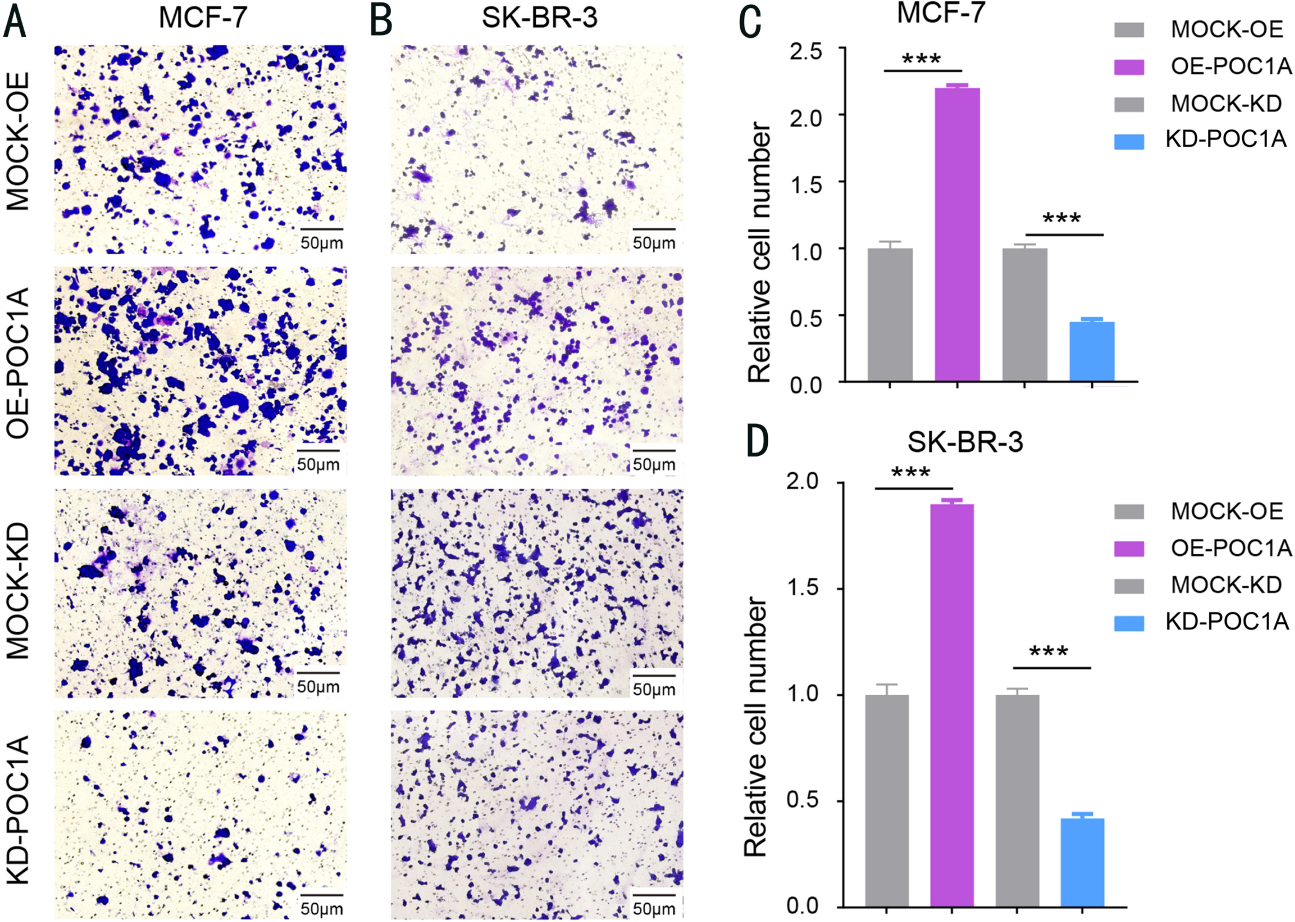
Effects of POC1A overexpression and knockdown on the invasion ability of breast cancer cells assessed by matrigel-transwell assay. **(A)** Representative images of Matrigel-Transwell invasion assays in MCF-7 cells with POC1A overexpression or knockdown. **(B)** Representative images of Matrigel-Transwell invasion assays in SK-BR-3 cells with POC1A overexpression or knockdown. MOCK-OE and MOCK-KD served as the corresponding negative controls for the overexpression and knockdown systems, respectively. Scale bar, 50 μm. **(C)** Quantitative analysis of invaded MCF-7 cells. **(D)** Quantitative analysis of invaded SK-BR-3 cells. Invasion ability was quantified by counting invaded cells in randomly selected microscopic fields, and the values were normalized to the corresponding MOCK control group. Data are presented as mean ± SD from three independent experiments. Statistical significance was determined using an unpaired two-tailed Student’s t-test for each indicated comparison. ***P < 0.001.

The effect of POC1A knockdown on cell invasion was evaluated using a Matrigel-coated Transwell invasion assay. Compared with the MOCK-KD group, the number of MCF-7 and SK-BR-3 cells penetrating the Matrigel matrix and migrating to the lower surface of the membrane was reduced in the KD-POC1A group. Quantitative analysis further demonstrated that the number of invading cells was significantly decreased in the KD-POC1A group in both cell lines (P < 0.001) (**Figure 11**).

#### 3.16.2 Effect of POC1A overexpression on cell invasion

The effect of POC1A overexpression on cell invasion was further examined. Compared with the MOCK-OE group, the OE-POC1A group exhibited increased numbers of MCF-7 and SK-BR-3 cells penetrating the Matrigel matrix. Quantitative analysis indicated that the number of invading cells was significantly higher in the OE-POC1A group than in the control group in both cell lines (P < 0.001) (**Figure 11**).

### 3.17 POC1A modulation is associated with alterations in Wnt/β-catenin signaling-related proteins and EMT-related markers

#### 3.17.1 POC1A overexpression is associated with increased Wnt/β-catenin signaling-related proteins and altered EMT-related marker expression

Compared with the control group, POC1A overexpression induced marked changes in the expression of key components of the Wnt/β-catenin signaling pathway in both cell lines.

In MCF-7 and SK-BR-3 cells, the OE-POC1A group exhibited increased expression of Wnt3a, β-catenin, and TCF7, along with elevated levels of phosphorylated GSK3β (p-GSK3β), whereas total GSK3β expression remained unchanged. In addition, Axin2, a negative-feedback regulator of the Wnt/β-catenin pathway, showed an increasing trend. Analysis of downstream target proteins further revealed increased expression of c-Myc and Cyclin D1 in the OE-POC1A group.

**Figure 12:**
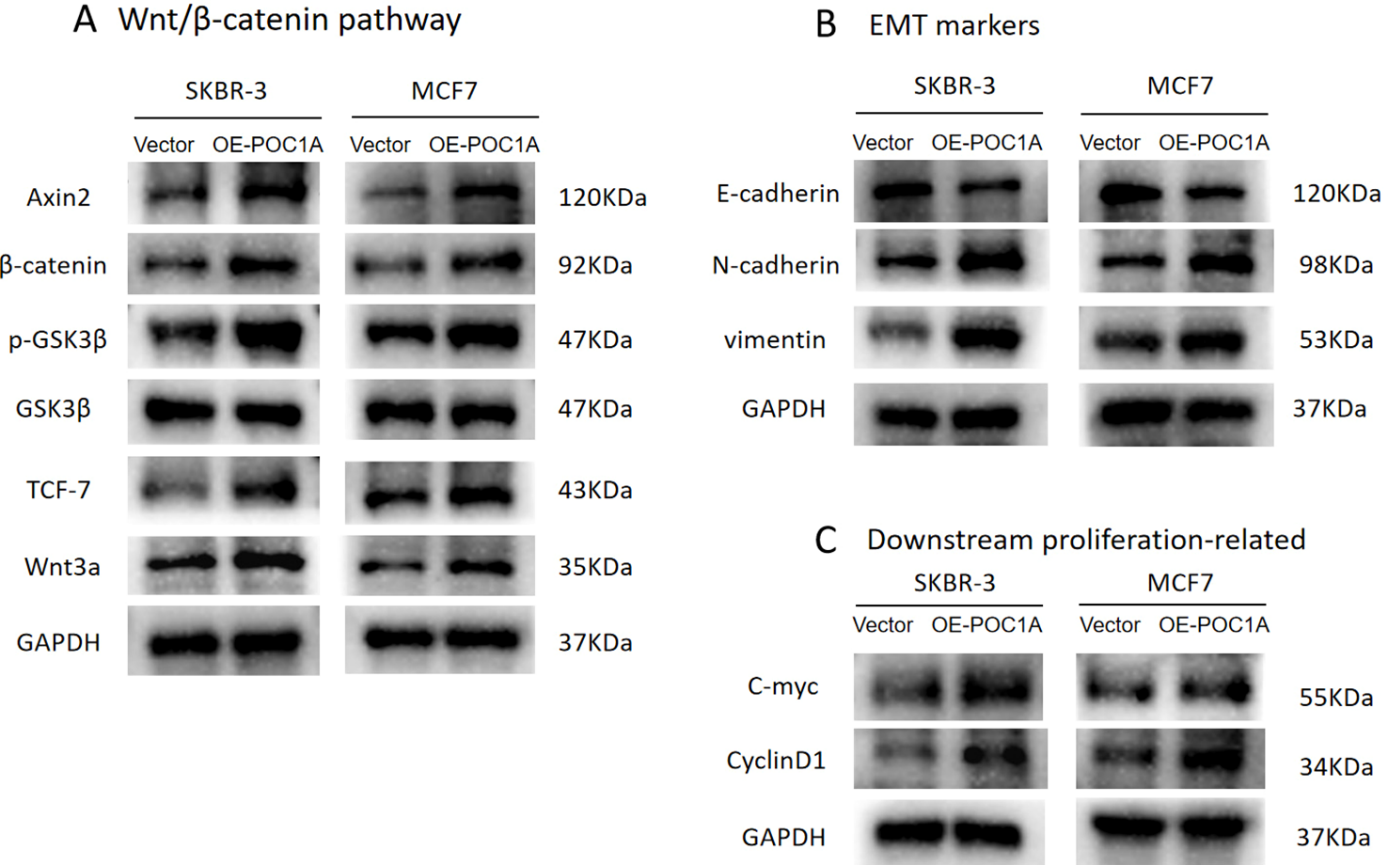
Western blot analysis of Wnt/β-catenin signaling-related proteins, epithelial-mesenchymal transition-related markers, and proliferation-related proteins after POC1A overexpression in breast cancer cells. **(A)** Representative Western blot images showing the expression of Wnt/β-catenin signaling-related proteins, including Axin2, β-catenin, phosphorylated GSK3β (p-GSK3β), total GSK3β, TCF7, and Wnt3a, in MCF-7 and SK-BR-3 cells after POC1A overexpression. Vector-transfected cells served as the corresponding control. GAPDH was used as the loading control. **(B)** Representative Western blot images showing the expression of epithelial-mesenchymal transition (EMT)-related markers, including E-cadherin, N-cadherin, and Vimentin, in MCF-7 and SK-BR-3 cells after POC1A overexpression. GAPDH was used as the loading control. **(C)** Representative Western blot images showing the expression of proliferation-related downstream proteins, including c-Myc and Cyclin D1, in MCF-7 and SK-BR-3 cells after POC1A overexpression. GAPDH was used as the loading control.

**Figure 13:**
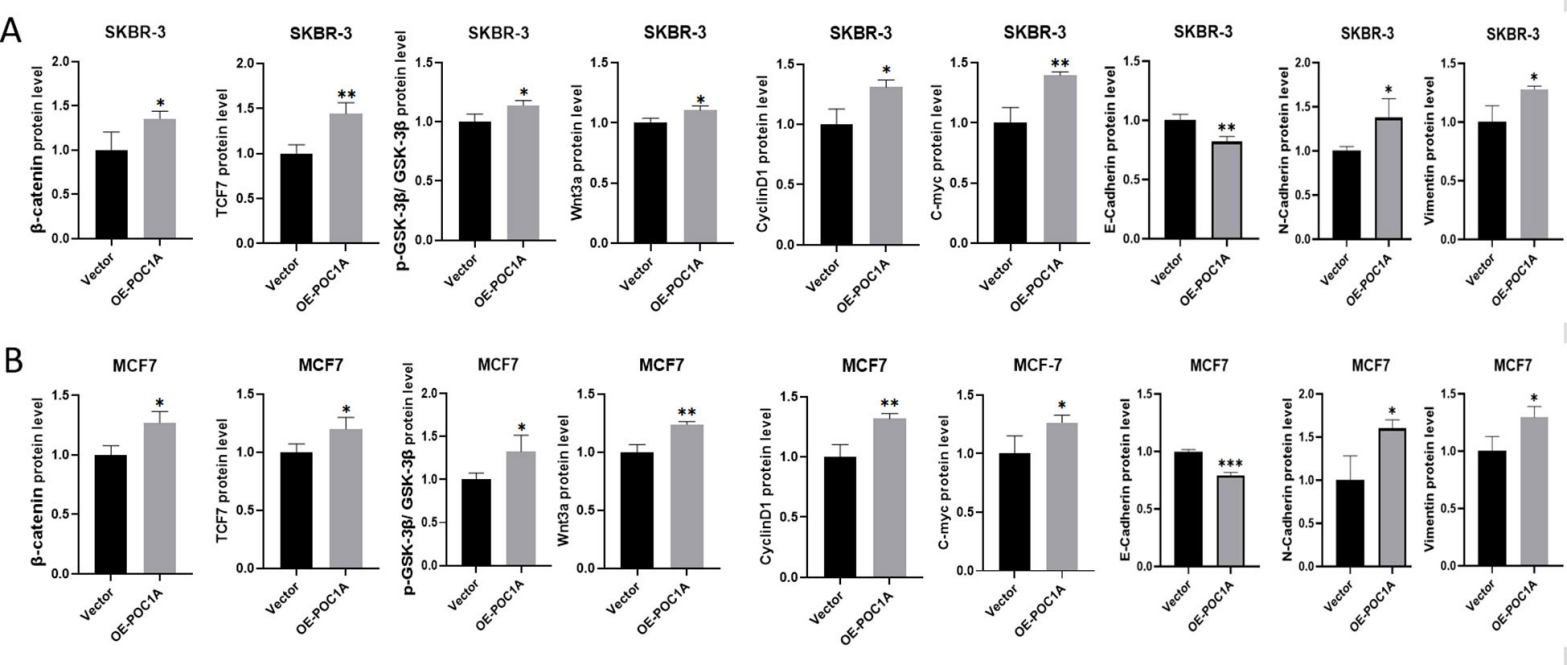
Densitometric quantification of Wnt/β-catenin signaling-related proteins, epithelial-mesenchymal transition-related markers, and proliferation-related proteins after POC1A overexpression in breast cancer cells. **(A)** Quantitative analysis of protein expression levels in SK-BR-3 cells after POC1A overexpression. **(B)** Quantitative analysis of protein expression levels in MCF-7 cells after POC1A overexpression. Densitometric values of β-catenin, TCF7, GSK3β, Wnt3a, Cyclin D1, c-Myc, E-cadherin, N-cadherin, and Vimentin were normalized to GAPDH, whereas phosphorylated GSK3β was normalized to total GSK3β. Data are presented as mean ± SD from three independent experiments. Statistical significance was determined using an unpaired two-tailed Student’s t-test for each indicated comparison. *P < 0.05, **P < 0.01, ***P < 0.001.

Concomitantly, EMT-related proteins showed consistent changes, with reduced E-cadherin expression and increased N-cadherin and Vimentin expression (**Figures 12**, **13**).

#### 3.17.2 POC1A knockdown is associated with reduced Wnt/β-catenin signaling-related proteins and reciprocal EMT-related marker changes

Conversely, POC1A knockdown resulted in marked alterations in the expression of Wnt/β-catenin pathway-related proteins in both cell lines compared with the control group.

In MCF-7 and SK-BR-3 cells, the si-POC1A group exhibited decreased expression of Wnt3a, β-catenin, and TCF7, along with reduced p-GSK3β levels, whereas total GSK3β expression remained largely unchanged. In addition, Axin2 expression also showed a decreasing trend in the si-POC1A group.

**Figure 14:**
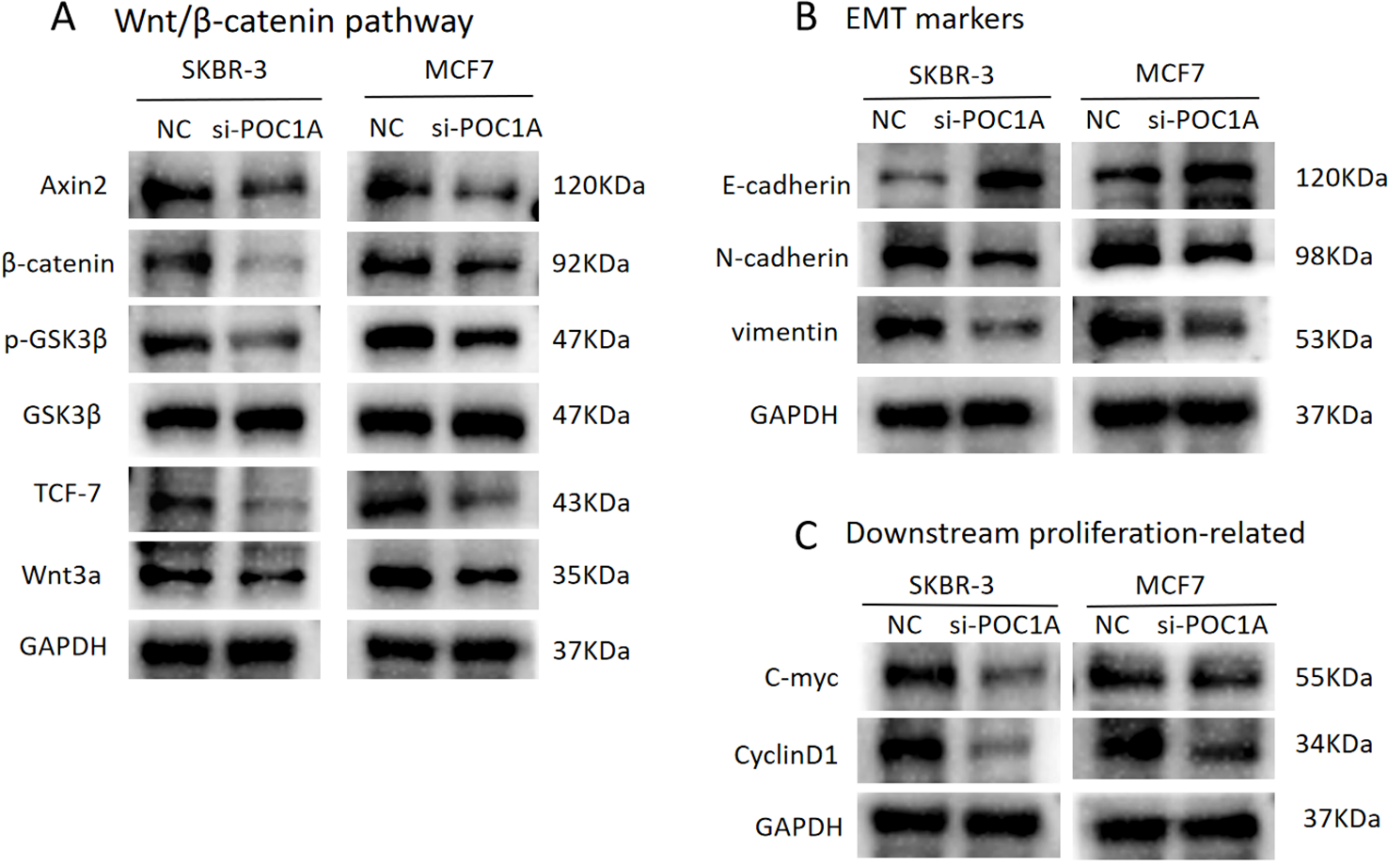
Western blot analysis of Wnt/β-catenin signaling-related proteins, epithelial-mesenchymal transition-related markers, and proliferation-related proteins after POC1A knockdown in breast cancer cells. **(A)** Representative Western blot images showing the expression of Wnt/β-catenin signaling-related proteins, including Axin2, β-catenin, phosphorylated GSK3β (p-GSK3β), total GSK3β, TCF7, and Wnt3a, in MCF-7 and SK-BR-3 cells after POC1A knockdown. NC served as the corresponding negative control. GAPDH was used as the loading control. **(B)** Representative Western blot images showing the expression of epithelial-mesenchymal transition (EMT)-related markers, including E-cadherin, N-cadherin, and Vimentin, in MCF-7 and SK-BR-3 cells after POC1A knockdown. GAPDH was used as the loading control. **(C)** Representative Western blot images showing the expression of proliferation-related downstream proteins, including c-Myc and Cyclin D1, in MCF-7 and SK-BR-3 cells after POC1A knockdown. GAPDH was used as the loading control.

**Figure 15:**
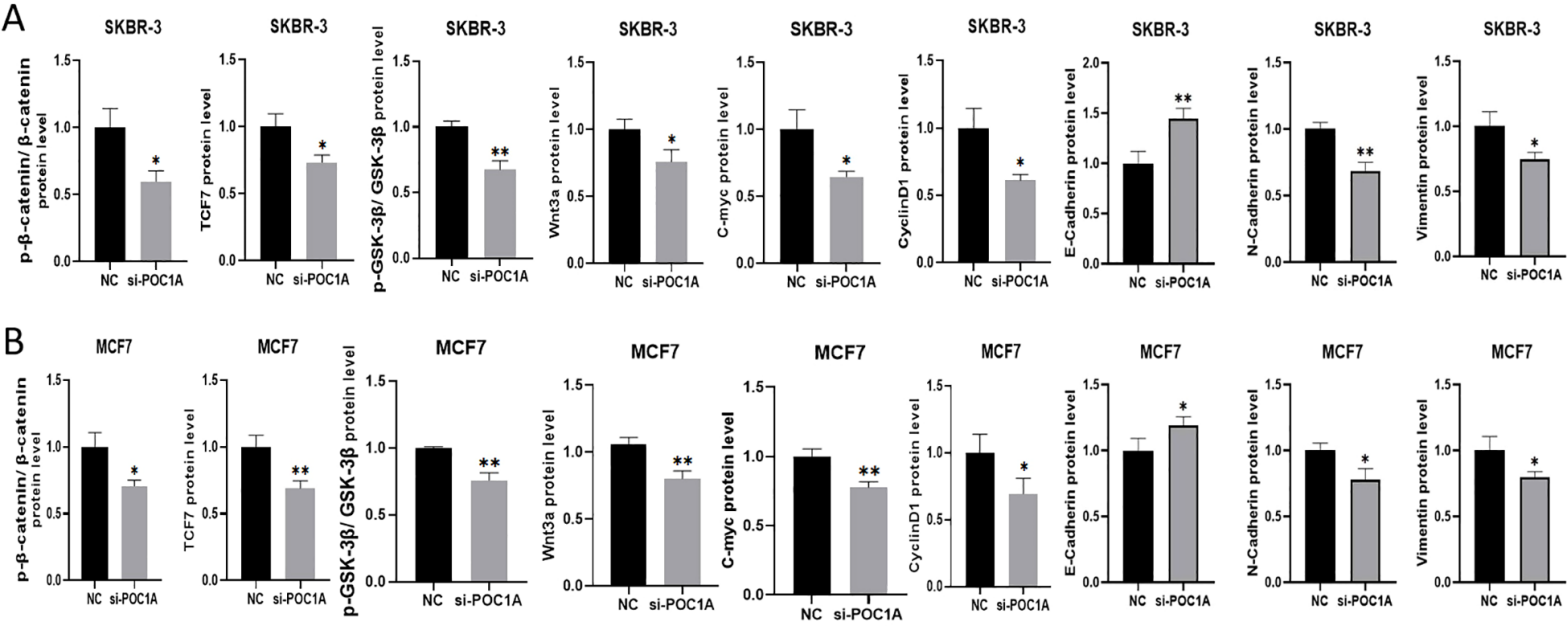
Densitometric quantification of Wnt/β-catenin signaling-related proteins, epithelial-mesenchymal transition-related markers, and proliferation-related proteins after POC1A knockdown in breast cancer cells. **(A)** Quantitative analysis of protein expression levels in SK-BR-3 cells after POC1A knockdown. **(B)** Quantitative analysis of protein expression levels in MCF-7 cells after POC1A knockdown. Densitometric values of β-catenin, TCF7, GSK3β, Wnt3a, c-Myc, Cyclin D1, E-cadherin, N-cadherin, and Vimentin were normalized to GAPDH, whereas phosphorylated GSK3β was normalized to total GSK3β. Data are presented as mean ± SD from three independent experiments. Statistical significance was determined using an unpaired two-tailed Student’s t-test for each indicated comparison. *P < 0.05, **P < 0.01.

Analysis of downstream targets showed that POC1A knockdown reduced the expression of c-Myc and Cyclin D1. In parallel, analysis of EMT-related proteins revealed increased E-cadherin expression and decreased N-cadherin and Vimentin expression in the si-POC1A group (**Figure 14**, **15**).

## 4 Discussion

This study systematically investigated the expression pattern, clinical relevance, biological functions, and potential molecular mechanisms of POC1A in breast cancer. Analyses of the TCGA and GEO public databases demonstrated that POC1A is significantly overexpressed in breast cancer tissues and is closely associated with clinicopathological features such as tumor size and clinical stage. Prognostic analysis of the TCGA cohort further revealed that elevated POC1A expression was significantly associated with unfavorable patient outcomes and remained an independent prognostic factor in multivariate Cox regression analysis. In addition, Kaplan–Meier Plotter analysis further supported the association between high POC1A mRNA expression and poor clinical outcomes in patients with breast cancer.

Immunohistochemical findings at the protein level corroborated the overexpression of POC1A in breast cancer tissues and indicated that higher expression was significantly associated with larger tumor size, higher histological grade, and more advanced clinical stage (P < 0.05). However, in the tissue microarray validation cohort, although POC1A protein expression was associated with prognosis in univariate Cox analysis, it did not retain independent prognostic significance in multivariate analysis. This discrepancy suggests that the prognostic value of POC1A should be interpreted cautiously and requires further validation at the protein level.

*In vitro* functional assays further demonstrated that POC1A promotes the proliferation, migration, and invasion of breast cancer cells. Integrating the results of GSEA and Western blot analyses, this study provides preliminary evidence that POC1A may contribute to the malignant progression of breast cancer, at least in part, through alterations in Wnt/β-catenin signaling-related proteins and EMT-related markers.

Importantly, Qian et al. recently reported that POC1A induces EMT and promotes growth and metastasis in TNBC through the STAT3 signaling pathway (16). This study provides important mechanistic evidence for the oncogenic role of POC1A in TNBC and identifies STAT3 as a key downstream pathway. Consistent with this, STAT3 has been widely recognized as an oncogenic signaling mediator in TNBC. A systematic review summarized that STAT3 is frequently overexpressed or constitutively activated in TNBC cells and contributes to cell survival, proliferation, cell-cycle progression, anti-apoptosis, migration, invasion, angiogenesis, chemoresistance, immunosuppression, and cancer stem cell-related properties (17).

Compared with the TNBC-focused study by Qian et al., the present study differs in cellular context and mechanistic emphasis. Our functional experiments were performed in MCF-7 and SK-BR-3 cells, which represent luminal and HER2-positive non-TNBC breast cancer models, respectively. Therefore, our findings should not be interpreted as replacing the previously reported POC1A–STAT3 mechanism. Rather, they suggest that POC1A may exert pro-tumorigenic effects in different breast cancer subtypes through context-dependent downstream signaling networks. It is possible that STAT3 signaling predominates in TNBC, whereas Wnt/β-catenin-related alterations may accompany POC1A-driven phenotypes in non-TNBC cellular contexts. However, this hypothesis remains preliminary because STAT3 activity was not directly examined in our models, and functional Wnt/β-catenin pathway assays were not performed. Future studies comparing STAT3 and Wnt/β-catenin signaling in TNBC, luminal, and HER2-positive models are required to determine whether these pathways act independently, sequentially, or cooperatively downstream of POC1A.

POC1A is a centrosome-associated protein that plays a pivotal role in centrosome assembly, spindle formation, and cell-cycle regulation (22) (23). Previous studies have shown that aberrant expression of POC1A is closely involved in the initiation and progression of various malignancies (10) (15) (24); however, its role in breast cancer has not been fully elucidated. In the present study, we confirmed the elevated expression of POC1A in breast cancer at both the transcriptional and protein levels, further supporting its close association with tumor progression. GSEA revealed that gene sets associated with high POC1A expression were predominantly enriched in pathways related to the cell cycle, DNA replication, oocyte meiosis, and oxidative phosphorylation, suggesting that POC1A may contribute to breast cancer progression by regulating proliferative and metabolic processes. GO enrichment analysis further suggested a potential role of POC1A in immune-related biological functions. These findings are largely consistent with previous reports implicating POC1A in cell-cycle regulation and tumor proliferation (25), thereby underscoring its potential value as a diagnostic and prognostic biomarker.

Although analysis of the TCGA cohort indicated that POC1A was associated with poor prognosis in patients with breast cancer and remained an independent prognostic risk factor in multivariate Cox regression analysis, findings from the tissue microarray validation cohort showed that POC1A protein expression was associated with patient outcomes only in univariate analysis and did not retain independent prognostic significance in multivariate analysis. This discrepancy may be attributed to several factors. First, the TCGA cohort includes a larger sample size, providing greater statistical power and increasing the reliability of detecting stable prognostic signals. In contrast, the tissue microarray validation cohort is relatively small, and the number of analyzable cases may be further limited by factors such as tissue loss, staining quality, and incomplete clinical data, thereby reducing statistical robustness (26). Second, the TCGA analysis was conducted at the transcriptomic level, whereas the immunohistochemical validation reflected protein expression; these two layers of biological regulation do not necessarily show complete concordance. Post-transcriptional modifications, translational efficiency, and protein degradation may all contribute to discrepancies between mRNA and protein expression levels (27).

Furthermore, commercial tissue microarrays typically sample only localized tumor regions, which may not adequately capture intratumoral heterogeneity and may therefore affect the concordance between immunohistochemical scoring and the overall expression status of the tumor. Therefore, although TCGA and Kaplan–Meier Plotter analyses suggest that POC1A may have prognostic relevance in breast cancer, its independent prognostic value at the protein level remains to be confirmed in larger, multicenter, and well-annotated clinical cohorts.

*In vitro* functional assays further support the oncogenic role of POC1A in breast cancer progression. Overexpression of POC1A enhanced cellular proliferation, colony formation, migration, and invasion, whereas its knockdown exerted the opposite effects, suggesting that POC1A contributes to the maintenance of malignant phenotypes in breast cancer cells.

Cell-cycle analysis further showed that modulation of POC1A expression was accompanied by changes in cell-cycle distribution. In both MCF-7 and SK-BR-3 cells, POC1A knockdown tended to increase the proportion of cells in S and/or G2/M phases, while reducing cell proliferation and colony formation. This pattern suggests that loss of POC1A may not simply slow proliferation by inducing a classical G0/G1 arrest, but may instead interfere with cell-cycle progression and lead to accumulation of cells at later cell-cycle phases. Given the known role of POC1A in centrosome function and mitotic regulation, these changes may reflect cell-cycle checkpoint activation or impaired progression through DNA replication and mitosis. Conversely, POC1A overexpression altered cell-cycle distribution in a cell-line-dependent manner, indicating that the effect of POC1A on cell-cycle regulation may depend on the intrinsic molecular background of breast cancer cells. Therefore, the cell-cycle results should be interpreted as supportive evidence that POC1A is associated with cell-cycle regulation, rather than as definitive proof of a specific cell-cycle checkpoint mechanism.

At the mechanistic level, this study established POC1A overexpression and knockdown models in MCF-7 and SK-BR-3 cells and examined the expression of key components of the Wnt/β-catenin signaling pathway as well as EMT-related molecules. The canonical Wnt/β-catenin pathway is an evolutionarily conserved signaling network involved in development, tissue homeostasis, and cancer-related biological processes; its activation is characterized by β-catenin stabilization, nuclear translocation, and TCF/LEF-mediated transcriptional regulation (18). The results showed that upregulation of POC1A was accompanied by coordinated increases in Wnt3a, β-catenin, TCF7, and downstream target proteins such as c-Myc and Cyclin D1, whereas POC1A knockdown led to an overall downregulation of these molecules, suggesting an association between POC1A expression and Wnt/β-catenin signaling-related alterations rather than definitively proving functional activation of this pathway.

Further analysis showed that POC1A overexpression resulted in increased levels of phosphorylated GSK3β (p-GSK3β), whereas total GSK3β expression remained largely unchanged. Concurrently, β-catenin, TCF7, and Axin2 showed an upward trend. Given that Axin2 is a canonical negative-feedback target of the Wnt/β-catenin pathway, its upregulation is generally indicative of pathway activation (28). Together with the concomitant changes in c-Myc and Cyclin D1, these findings suggest that POC1A may be associated with alterations in Wnt/β-catenin-related downstream proteins, but they do not provide sufficient direct evidence to establish Wnt/β-catenin signaling as a confirmed functional mediator of POC1A-driven malignant phenotypes.

Moreover, the β-catenin/TCF-mediated transcriptional network is known to participate in EMT-associated transcriptional regulation and remodeling of cell-adhesion molecules, thereby weakening intercellular epithelial junctions and enhancing cytoskeletal dynamics, migratory capacity, and invasive potential (29). Previous reviews have also indicated that dysregulation of Wnt/β-catenin signaling can promote EMT, a process characterized by β-catenin nuclear translocation, E-cadherin suppression, and transcriptional activation of EMT-related genes such as Twist and Snail through TCF/LEF-related programs (30). In the present study, POC1A overexpression was associated with a characteristic EMT-related molecular profile, including downregulation of E-cadherin and upregulation of N-cadherin and Vimentin, whereas the opposite pattern was observed following POC1A knockdown. These results indicate that POC1A may be associated with EMT-related molecular reprogramming in breast cancer cells. However, because direct functional assays of Wnt/β-catenin activity, such as β-catenin nuclear translocation, TCF/LEF reporter assays, and Wnt pathway inhibition or rescue experiments, were not performed, the relationship among POC1A, Wnt/β-catenin signaling, and EMT should be regarded as preliminary and hypothesis-generating.

Taken together, our findings suggest a working model in which POC1A promotes malignant phenotypes in non-TNBC breast cancer cell models and is accompanied by alterations in Wnt/β-catenin signaling-related proteins and EMT-related markers. This model differs from, but does not contradict, the TNBC-focused POC1A–STAT3 axis reported by Qian et al. Instead, these findings collectively suggest that POC1A may participate in breast cancer progression through subtype-dependent downstream signaling networks. Future studies directly comparing STAT3 and Wnt/β-catenin signaling in TNBC and non-TNBC models will be important to clarify the molecular context of POC1A function in breast cancer.

This study has several limitations. First, analyses based on public databases relied on retrospective data and may therefore be susceptible to selection bias. Second, the tissue microarray validation cohort was relatively small, and its reliance on commercially sourced specimens may have affected both statistical power and the generalizability of the findings. Third, although Western blot analysis showed changes in Wnt/β-catenin signaling-related proteins, direct evidence of pathway activation remains lacking. More direct experiments, such as assessment of β-catenin nuclear translocation, TCF/LEF reporter assays, and pathway inhibition or rescue experiments, are still needed. Fourth, the present study did not examine STAT3 signaling or determine whether the effects observed in MCF-7 and SK-BR-3 cells are independent of the STAT3 pathway. Given the recent findings of Qian et al. in TNBC, future studies should further investigate the crosstalk between STAT3 and Wnt/β-catenin signaling in different breast cancer subtypes. Therefore, whether POC1A promotes breast cancer progression through a Wnt/β-catenin–EMT-related mechanism warrants further in-depth investigation.

## 5 Conclusion

In conclusion, POC1A is overexpressed in breast cancer and may contribute to malignant progression by promoting cell proliferation, migration, and invasion. In contrast to the previously reported POC1A–STAT3 mechanism in TNBC, the present study examined POC1A function in MCF-7 and SK-BR-3 non-TNBC breast cancer cell models and observed concomitant changes in Wnt/β-catenin signaling-related proteins and EMT-related markers. These findings suggest that POC1A may participate in breast cancer progression through subtype-dependent molecular networks. However, direct functional validation of Wnt/β-catenin pathway involvement and further clarification of the relationship between POC1A and STAT3 signaling are still required. POC1A may represent a candidate molecular marker and a potential target for further investigation in breast cancer, although its diagnostic, prognostic, and mechanistic significance requires validation in larger clinical cohorts and mechanistic models.

## Data Availability

The original contributions presented in the study are included in the article/**Supplementary Material**. Further inquiries can be directed to the corresponding author.

## Supplementary material

Supplementary Figure 1 Association of POC1A expression with clinicopathological features of breast cancer. **(A)** POC1A expression across different T stages. **(B)** POC1A expression across different N stages. **(C)** POC1A expression across different M stages. **(D)** POC1A expression across different clinical stages. **(E)** POC1A expression across different age groups. **(F)** POC1A expression by sex. p-values indicate intergroup differences. T, N, M: TNM staging; Stage: clinical stage.

Supplementary Figure 2 Construction and validation of the nomogram model. **(A)** Nomogram constructed based on multivariate Cox regression analysis to predict 1-, 3-, and 5-year overall survival (OS) probabilities in breast cancer patients. **(B)** ROC curve evaluating the discriminative ability of the nomogram. **(C)** Calibration curves assessing the agreement between predicted and observed survival probabilities of the nomogram model.

Supplementary Figure 3 Forest plot of univariate and multivariate Cox regression analyses based on POC1A. **(A)** Univariate Cox regression analysis assessing the association of each variable with overall survival (OS). **(B)** Multivariate Cox regression analysis including variables with statistical significance in the univariate analysis to evaluate their independent prognostic value.

Supplementary Figure 4 Expression of POC1A in breast cancer cell lines and normal mammary epithelial cells. **(A)** qRT-PCR analysis of POC1A mRNA expression in the normal mammary epithelial cell line MCF-10A and breast cancer cell lines MDA-MB-231, MCF-7, and SK-BR-3. **(B)** Quantitative analysis of POC1A protein levels in MCF-10A and breast cancer cell lines by Western blot. **(C)** Representative Western blot bands of POC1A protein, with GAPDH as a loading control. *P < 0.05, **P < 0.01, ***P < 0.001.

Supplementary Figure 5 Validation of POC1A overexpression and knockdown efficiency in breast cancer cells. **(A)** qRT-PCR analysis of POC1A mRNA expression in MCF-7 cells with overexpression (OE), knockdown (KD), and negative control (NC). **(B)** qRT-PCR analysis of POC1A mRNA expression in SK-BR-3 cells with overexpression (OE), knockdown (KD), and negative control (NC). Data were normalized to GAPDH and are presented as relative expression levels. ***P < 0.001.

Supplementary Figure 6 Validation of POC1A overexpression and knockdown at the protein level in breast cancer cells. **(A)** Western blot analysis of POC1A protein expression in SK-BR-3 and MCF-7 cells with overexpression (OE), knockdown (KD), and negative control (NC), using GAPDH as a loading control. **(B)** Quantitative analysis of POC1A protein relative expression in MCF-7 cells based on band intensity. **(C)** Quantitative analysis of POC1A protein relative expression in SK-BR-3 cells based on band intensity. Data were normalized to GAPDH and are presented as relative expression levels. ***P < 0.001.

Supplementary Figure 7 Available western blot source images for MCF-7 cells after POC1A knockdown. Available western blot source images showing Wnt/β-catenin signaling-related proteins, proliferation-related proteins, and epithelial-mesenchymal transition (EMT)-related markers in MCF-7 cells after POC1A knockdown. NC served as the negative control, and si-POC1A represented the POC1A knockdown group. GAPDH bands correspond to the same sample sets and lane order as the indicated target proteins. Three independent biological replicates are shown.

Supplementary Figure 8 Available western blot source images for MCF-7 cells after POC1A overexpression. Available Western blot source images showing Wnt/β-catenin signaling-related proteins, proliferation-related proteins, and EMT-related markers in MCF-7 cells after POC1A overexpression. Vector-transfected cells served as the control, and OE-POC1A represented the POC1A overexpression group. GAPDH bands correspond to the same sample sets and lane order as the indicated target proteins. Three independent biological replicates are shown.

Supplementary Figure 9 Available western blot source images for SK-BR-3 cells after POC1A knockdown. Available Western blot source images showing Wnt/β-catenin signaling-related proteins, proliferation-related proteins, and EMT-related markers in SK-BR-3 cells after POC1A knockdown. NC served as the negative control, and si-POC1A represented the POC1A knockdown group. GAPDH bands correspond to the same sample sets and lane order as the indicated target proteins. Three independent biological replicates are shown.

Supplementary Figure 10 Available western blot source images for SK-BR-3 cells after POC1A overexpression. Available western blot source images showing Wnt/β-catenin signaling-related proteins, proliferation-related proteins, and EMT-related markers in SK-BR-3 cells after POC1A overexpression. Vector-transfected cells served as the control, and OE-POC1A represented the POC1A overexpression group. GAPDH bands correspond to the same sample sets and lane order as the indicated target proteins. Three independent biological replicates are shown.

Supplementary Table 1 Primers of human POC1A and ACTB.

Supplementary Table 2 Antibodies used in this study.

## Glossary

POC1A: proteome of centriole 1A
EMT: epithelial-mesenchymal transition
ROC: receiver operating characteristic
AUC: area under the curve
CI: confidence interval
HR: hazard ratio
C-index: concordance index
GSEA: gene set enrichment analysis
log2FC: log2 fold change
KEGG: Kyoto Encyclopedia of Genes and Genomes
GO: Gene Ontology
BP: biological process
CC: cellular component
MF: molecular function
NES: normalized enrichment score
FDR: false discovery rate
DMEM: Dulbecco’s modified Eagle medium
FBS: fetal bovine serum
si-NC: negative control siRNA
qRT-PCR: quantitative real-time polymerase chain reaction
PBS: phosphate-buffered saline
OE: overexpression
KD: knockdown
NC: negative control
OD: optical density
CCK-8: Cell Counting Kit-8. ROC, receiver operating characteristic
AUC: area under the curve
CI: confidence interval
HRs: hazard ratios
C-index: concordance index
GSEA: gene set enrichment analysis
log2FC: log2 fold change
KEGG: Kyoto Encyclopedia of Genes and Genomes
GO: Gene Ontology
BP: biological process
CC: cellular component
MF: molecular function
NES: normalized enrichment score
FDR: false discovery rate
DMEM: Dulbecco’s modified Eagle medium
FBS: fetal bovine serum
Si-NC: negative control siRNA;qRT-PCR:Quantitative real-time polymerase chain reaction
PBS: prechilled phosphate-buffered saline
OE: overexpression group
KD: knockdown
NC: negative control
OD: optical density
CCK-8: Cell Counting Kit-8
STAT3: signal transducer and activator oftranscription 3
