# Supplementary figures and images for: POC1A promotes malignant phenotypes in non-triple-negative breast cancer cell models with EMT- and Wnt/β-catenin-related alterations
Source: Front Oncol. 2026 Jun 9;16:1856788. doi: 10.3389/fonc.2026.1856788 (PMC13286842; doi:10.3389/fonc.2026.1856788)

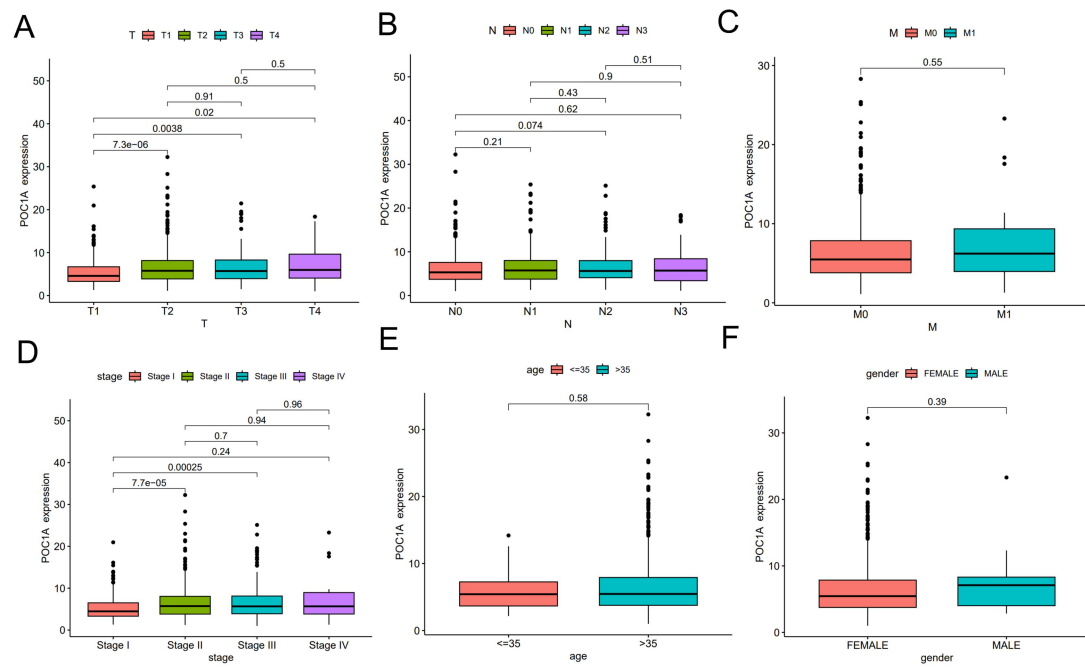

**Figure S1**

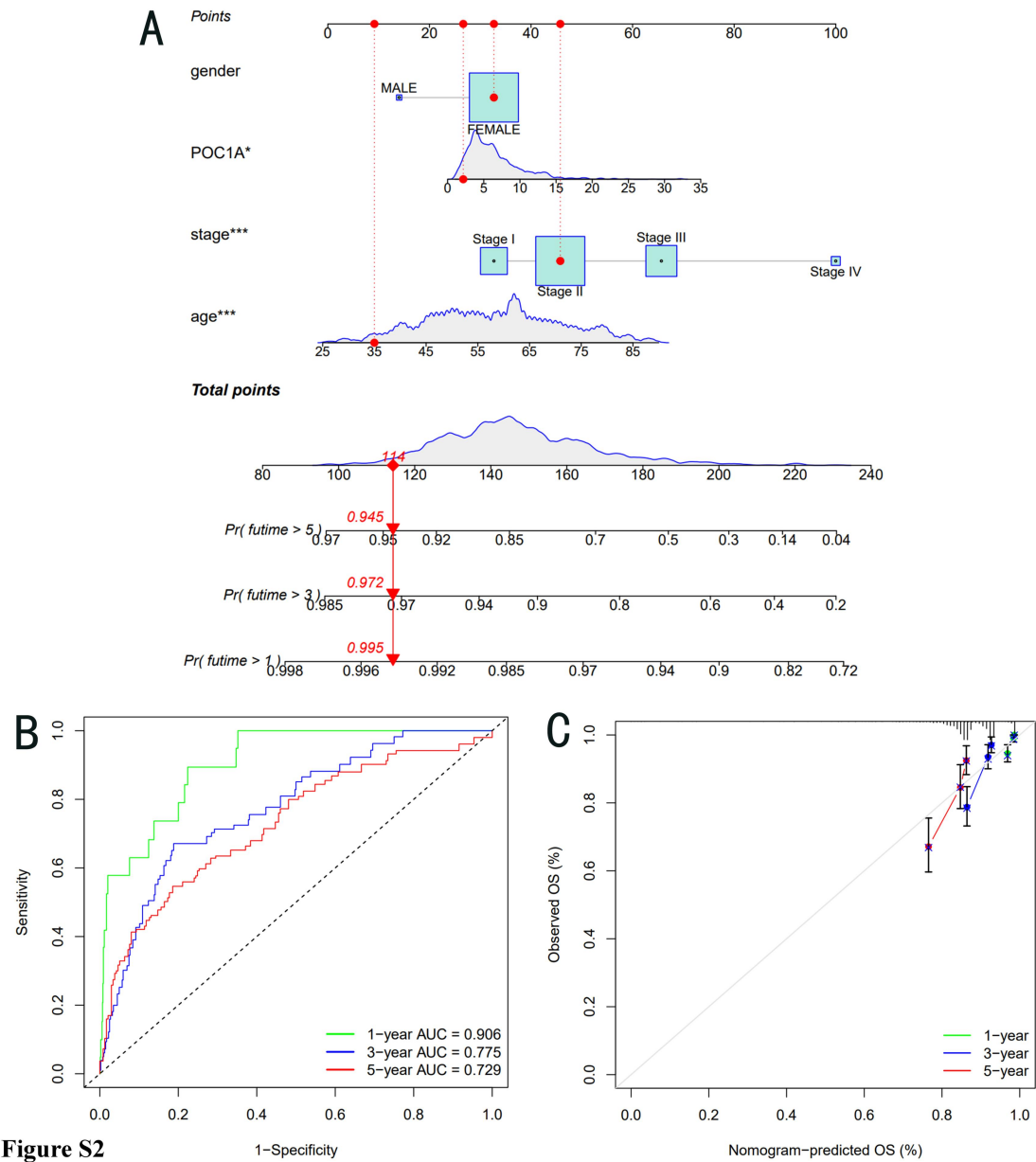

**Figure S2**

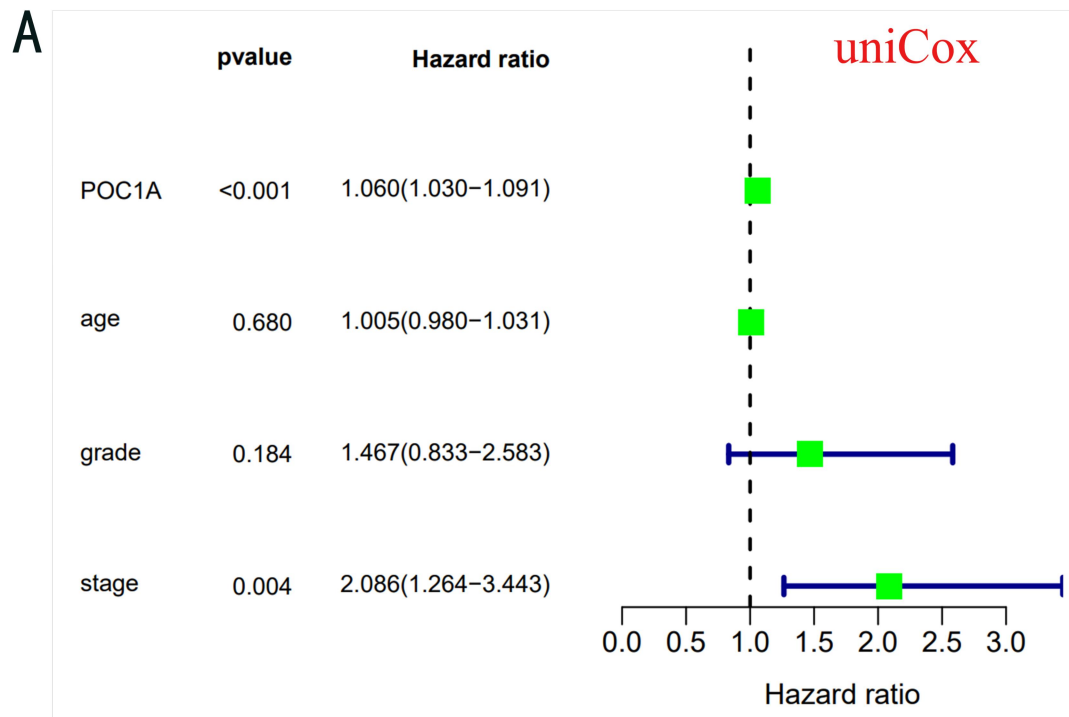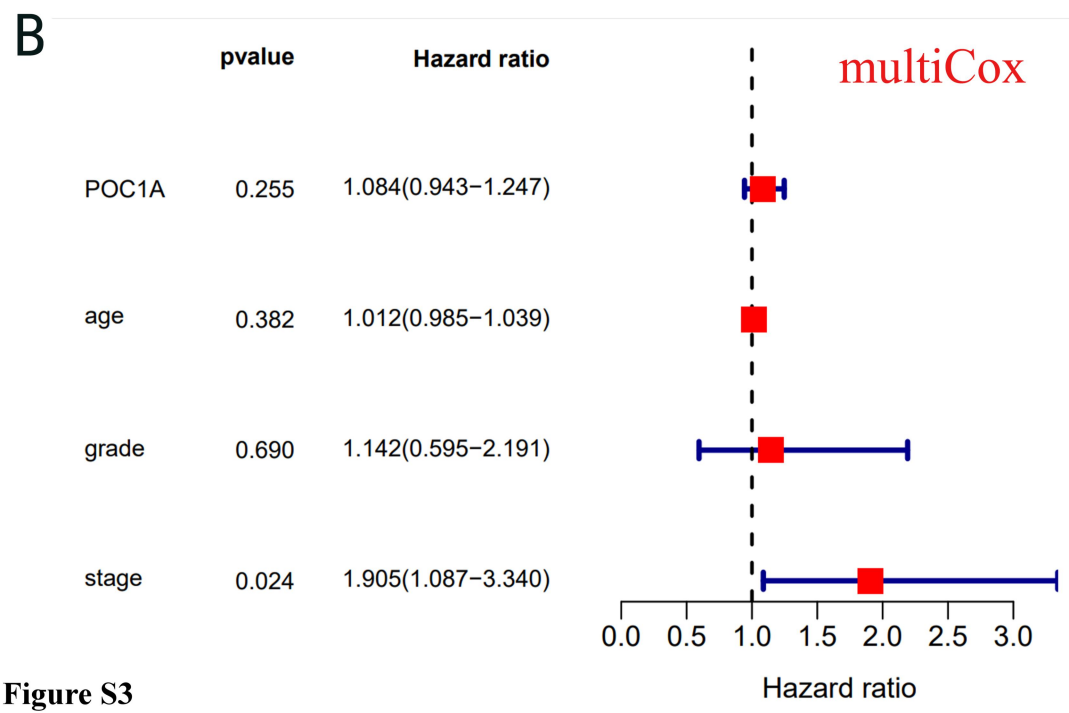

**Figure S3**

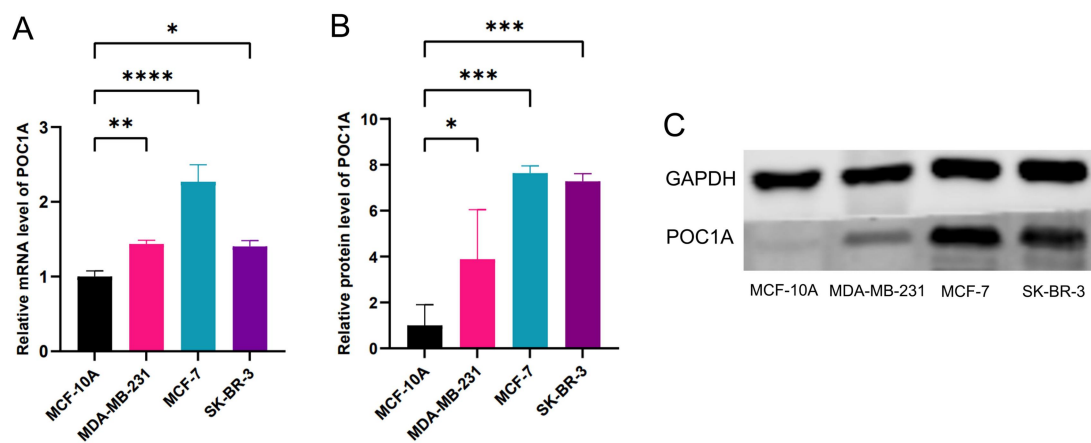

Figure S4

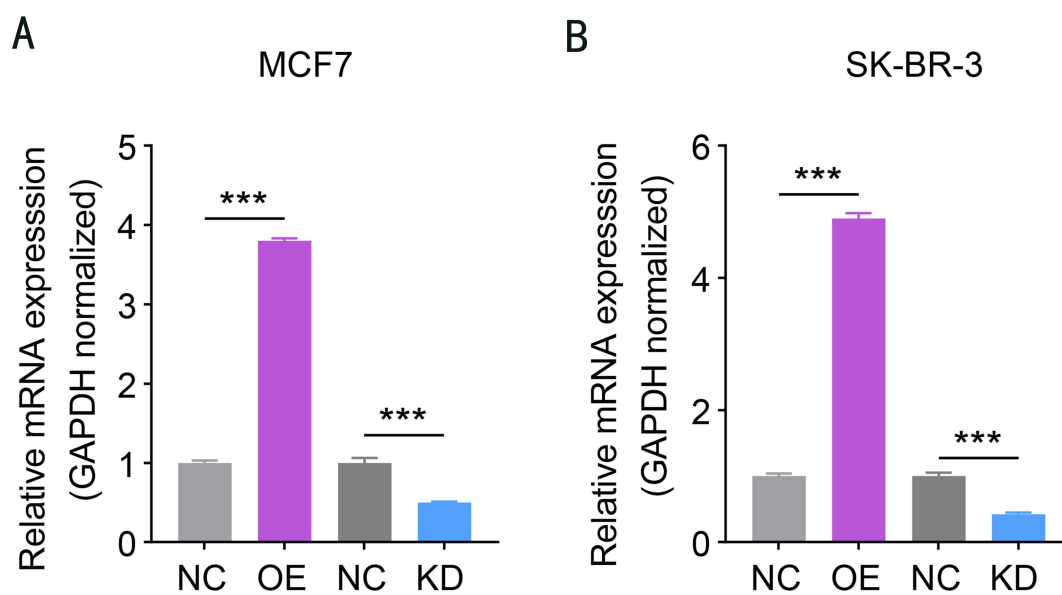

Figure S5

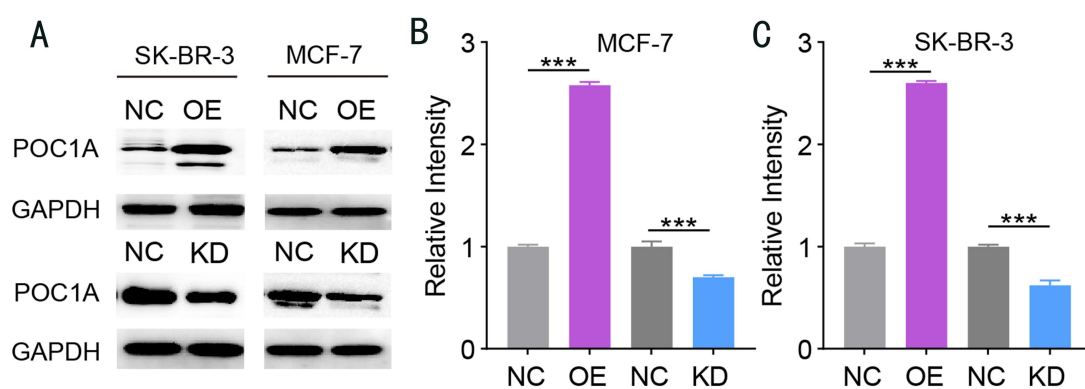

Figure S6

Supplement: Supplementary Figure 1 — Association of POC1A expression with clinicopathological features of breast cancer. (A) POC1A expression across different T stages. (B) POC1A expression across different N stages. (C) POC1A expression across different M stages. (D) POC1A expression across different clinical stages. (E) POC1A expression across different age groups. (F) POC1A expression by sex. p-values indicate intergroup differences. T, N, M: TNM staging; Stage: clinical stage. [file DataSheet1.pdf]
