# Supplementary material for: POC1A promotes malignant phenotypes in non-triple-negative breast cancer cell models with EMT- and Wnt/β-catenin-related alterations
Source: Front Oncol. 2026 Jun 9;16:1856788. doi: 10.3389/fonc.2026.1856788 (PMC13286842; doi:10.3389/fonc.2026.1856788)
Supplement: Supplementary file 2 [file DataSheet2.pdf]

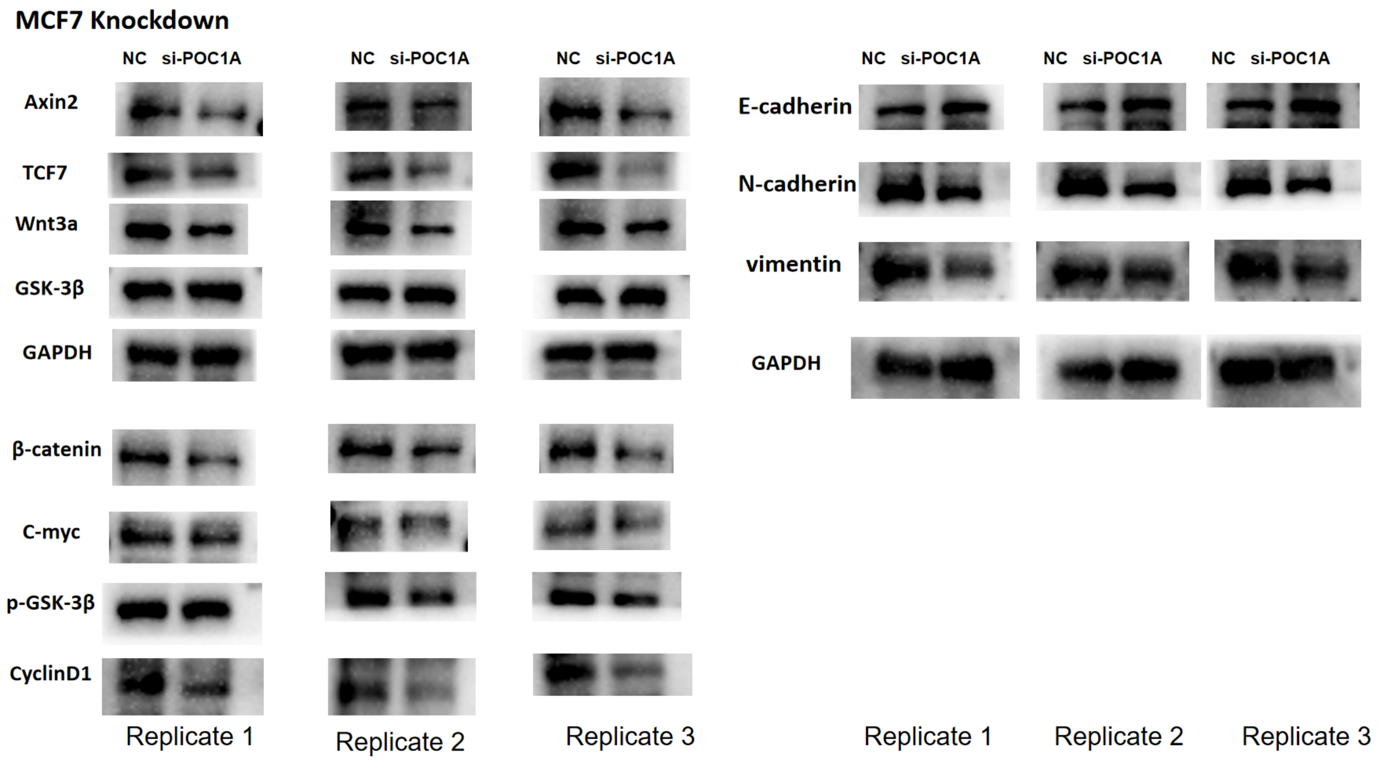
MCF7 Knockdown
NC si-POC1A
NC si-POC1A
NC si-POC1A
Axin2
TCF7
Wnt3a
GSK-3β
GAPDH
β-catenin
C-myc
p-GSK-3β
CyclinD1
Replicate 1
Replicate 2
Replicate 3
NC si-POC1A
NC si-POC1A
NC si-POC1A
E-cadherin
N-cadherin
vimentin
GAPDH
Replicate 1
Replicate 2
Replicate 3

Figure S7

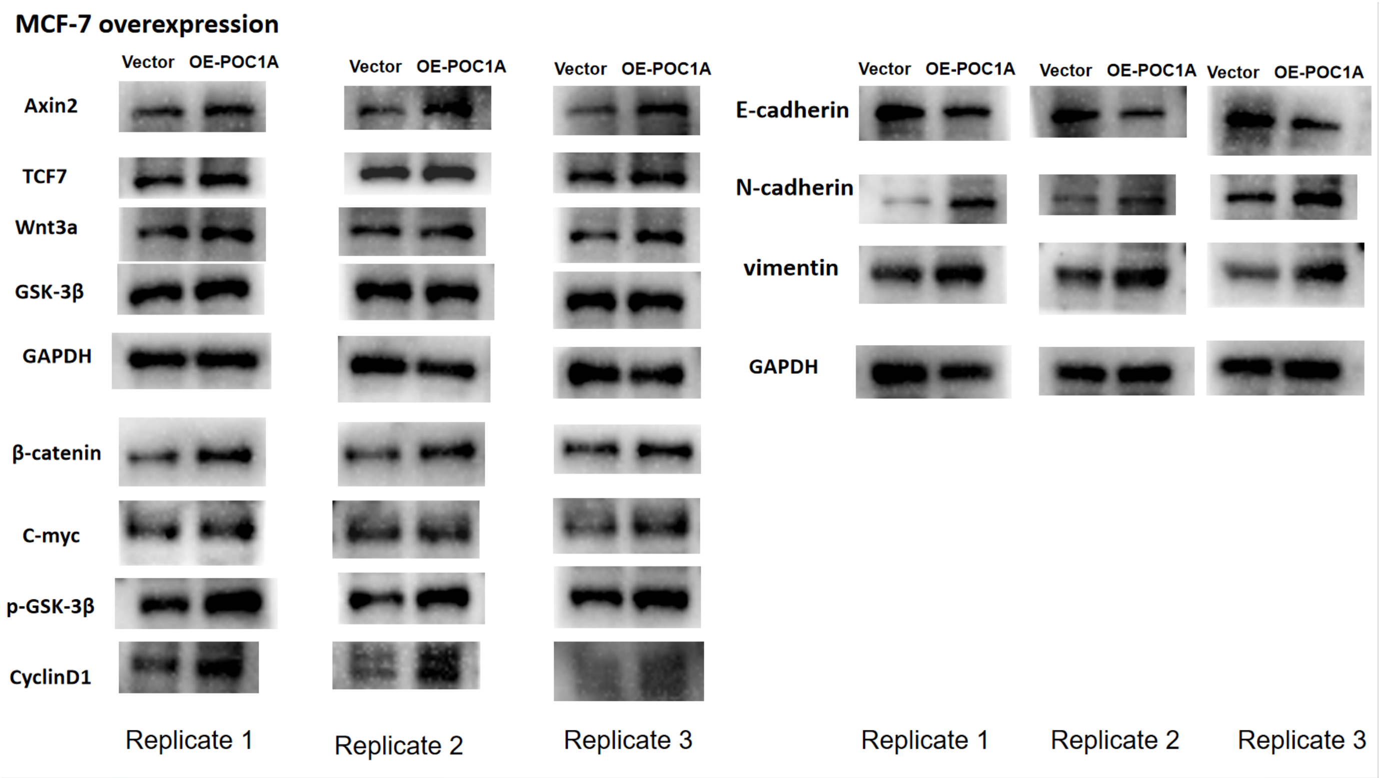
MCF-7 overexpression
Vector
OE-POC1A
Axin2
TCF7
Wnt3a
GSK-3β
GAPDH
β-catenin
C-myc
p-GSK-3β
CyclinD1
E-cadherin
N-cadherin
vimentin
Replicate 1
Replicate 2
Replicate 3

Figure S8

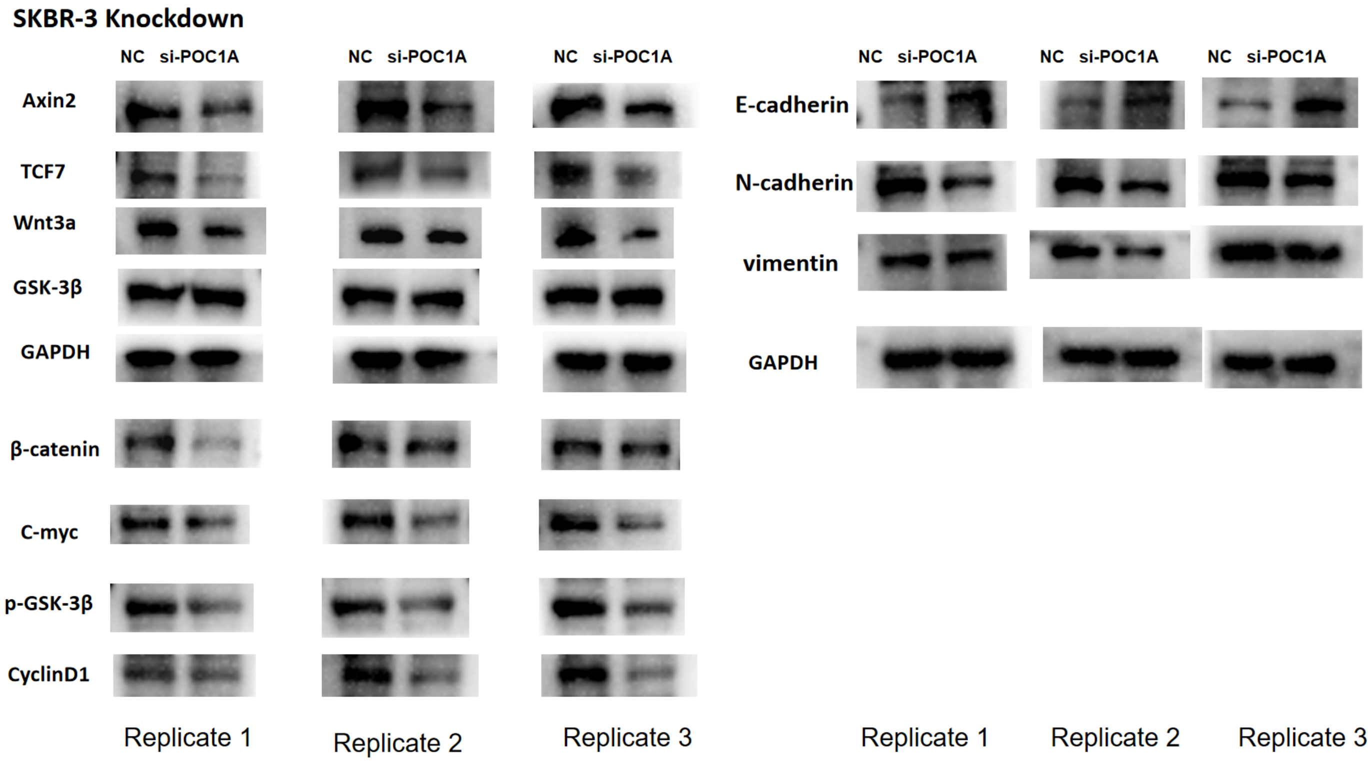
SKBR-3 Knockdown
NC
si-POC1A
Axin2
TCF7
Wnt3a
GSK-3β
GAPDH
β-catenin
C-myc
p-GSK-3β
CyclinD1
E-cadherin
N-cadherin
vimentin
Replicate 1
Replicate 2
Replicate 3

Figure S9

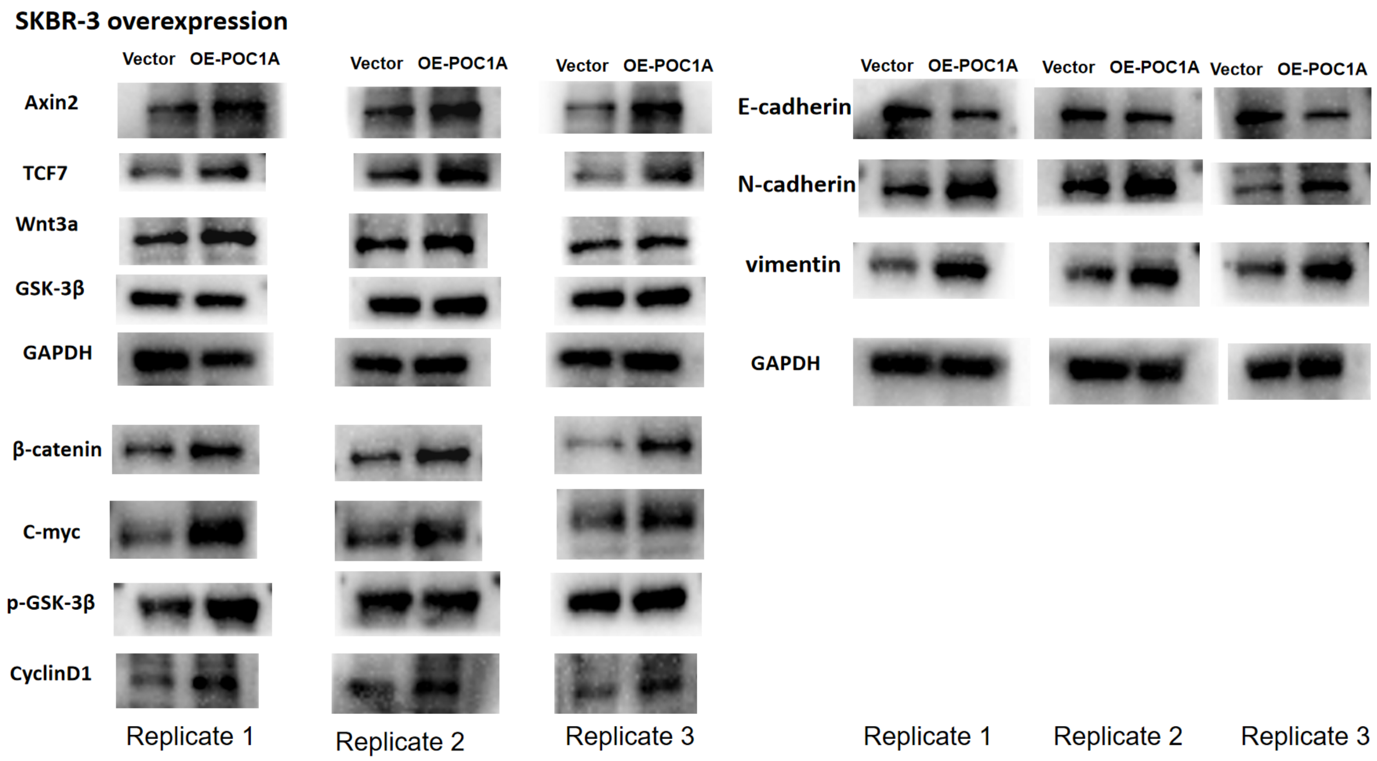
SKBR-3 overexpression
Vector
OE-POC1A
Axin2
TCF7
Wnt3a
GSK-3β
GAPDH
β-catenin
C-myc
p-GSK-3β
CyclinD1
E-cadherin
N-cadherin
vimentin
Replicate 1
Replicate 2
Replicate 3

Figure S10
