# Supplementary material for: POC1A promotes malignant phenotypes in non-triple-negative breast cancer cell models with EMT- and Wnt/β-catenin-related alterations
Source: Front Oncol. 2026 Jun 9;16:1856788. doi: 10.3389/fonc.2026.1856788 (PMC13286842; doi:10.3389/fonc.2026.1856788)
Supplement: Supplementary file 4 [file Table1.docx]

Table S1 Primers of human POC1A and β-actin

| Genes | Primers | Sequences | Product length |
| --- | --- | --- | --- |
| POC1A | Forward | GTCCGCTGTGCCAAGTTCTC | 124 |
| POC1A | Reverse | AGCCGCCATGCTCACAATAC |  |
| β-actin | Forward | TCACCATGGATGATGATATCGC | 150 |
| β-actin | Reverse | CTTCCCCACATAGGAATCCTTCTGACC |  |

Table S2 The antibodies used in this study.

| **Antibody** | **Supplier** | **Catalog No.** | **Dilution** | **Expected molecular weight (kDa)** |
| --- | --- | --- | --- | --- |
| POC1A | Zhengneng | 826521 | 1:1000 | 45 |
| β-catenin | Wanleibio | WL0962a | 1:1000 | 100 |
| c-Myc | ABclonal | A19032 | 1:1000 | 55 |
| TCF7 | Proteintech | 14464-1-AP | 1:1000 | 43 |
| Cyclin D1 | Proteintech | 60186-1-Ig | 1:5000 | 34 |
| GSK3β | Wanleibio | WL01456 | 1:1000 | 47 |
| Phospho-GSK3β | Wanleibio | WL03683 | 1:1000 | 47 |
| GAPDH | ABclonal | 81640-5-RR | 1:6000 | 37 |
| Wnt3a | Wanleibio | WL02179 | 1:1000 | 35 |
| Axin2 | ABclonal | A2513 | 1:2000 | 120 |
| HRP-conjugated Goat anti-Rabbit IgG (H+L) | ABclonal | AS014 | 1:10000 | — |
